# eIF3e-mediated translational checkpoint maintains immune tolerance and prevents lymphoid malignancy

**DOI:** 10.1084/jem.20251968

**Published:** 2026-06-03

**Authors:** Lianghua Lin, Pengda Chen, Jiazhen Wang, Chao Huang, Xinyong Lin, Chenfeng Liu, Kunyu Liao, Jianfeng Wu, Amy Chadburn, Yunjie Zhang, Huilin Song, Chenxi Wang, Zhichen Wan, Jiayi Zhao, Guangyi Cao, Xinming Wang, Liang Yang, Nan Yao, Yazhen Hong, Zhengtao Xiao, Dieter A. Wolf, Wen-Hsien Liu, Changchun Xiao

**Affiliations:** 1 State Key Laboratory of Cellular Stress Biology, School of Life Sciences, Faculty of Medicine and Life Sciences, Xiamen University, Xiamen, China; 2 Division of Hematology and Oncology, Department of Medicine and Meyer Cancer Center, Weill Cornell Medicine, New York, NY, USA; 3 Department of Biochemistry and Molecular Biology, School of Basic Medical College, Xi’an Jiaotong University, Xi’an, China; 4 Department of Cell Biology, School of Life Science, Anhui Medical University, Hefei, China; 5 Department of Pathology and Laboratory Medicine, Weill Cornell Medicine, New York, NY, USA; 6 Key Laboratory of Growth Regulation and Translational Research of Zhejiang Province, School of Life Sciences, Westlake University, Hangzhou, China; 7 Westlake Laboratory of Life Sciences and Biomedicine, School of Medicine, Westlake University, Hangzhou, China; 8 Department of Immunology and Microbiology, The Scripps Research Institute, La Jolla, CA, USA

## Abstract

Translational control is essential for immune function, but its roles in immune tolerance and lymphomagenesis remain poorly defined. Here, we show that *Cγ1*^Cre^-mediated deletion of *Eif3e*, which encodes a subunit of the eIF3 translation initiation complex, in B cells causes lymphoproliferation, malignant transformation of *Eif3e*-sufficient bystander lymphocytes, and premature death. *Eif3e*-deficient B cells upregulate the costimulatory molecule CD80, promoting CD4^+^ T cell activation and differentiation into IL-4–producing T_FH_-like cells. These cells, in turn, activate bystander B cells, increase MHC class II expression, and establish a feedforward loop of *Eif3e* deletion and lymphocyte activation. Despite their hyperactivated state, *Eif3e*-deficient B cells exhibit impaired proliferation, reduced survival, and defective differentiation. This self-amplifying circuit of aberrant B and T cell activation ultimately drives malignant transformation of *Eif3e*-sufficient lymphocytes. Our findings uncover an eIF3e-dependent translational checkpoint that preserves immune homeostasis and restrains lymphomagenesis.

## Teaser

Lin et al. identify eIF3e as a translational checkpoint that maintains immune tolerance. Deleting eIF3e in B cells drives pathological B–T cell interactions, lymphoproliferative disease, and lymphoma, highlighting translational control as a critical mechanism protecting against immune dysregulation and cancer.

## Introduction

During lymphocyte development, central tolerance mechanisms such as clonal deletion and receptor editing effectively eliminate most newly generated self-reactive B and T cells in the bone marrow and thymus, particularly those expressing antigen receptors with high affinity for self-antigens or self-peptides presented by MHC molecules. However, a substantial proportion of self-reactive lymphocytes escape central tolerance and persist in the periphery. Those cells are normally held in check by multiple peripheral tolerance mechanisms, including anergy and regulatory T (Treg) cell–mediated suppression. Disruption of these regulatory pathways, through aberrant signaling, impaired cytokine production, or defective Treg function, can lead to the breakdown of immune tolerance and the development of autoimmune disease. Although transcriptional, epigenetic, and signaling regulators of immune homeostasis have been extensively studied (Goodnow, 2007; Rawlings et al., 2017; Tanaka et al., 2020), whether translational control directly maintains immune tolerance *in vivo* remains unknown.

eIF3 is the largest and most structurally complex translation initiation factor in mammals, consisting of 13 subunits (eIF3a–m). It serves both as a scaffold that bridges the eIF4F and translation preinitiation complexes and as a regulatory hub for translational control. Beyond its canonical role in cap-dependent initiation, emerging evidence indicates that eIF3 also mediates noncanonical translation initiation via internal ribosome entry sites, m^6^A-modified transcripts, and other cellular context–specific mechanisms. Notably, individual eIF3 subunits have been implicated in specialized regulatory functions (Herrmannová et al., 2024; Hinnebusch, 2006; Lamper et al., 2020; Lee et al., 2015; Morris et al., 2007; Valášek et al., 2017; Wolf et al., 2020). In a recent CRISPR/Cas9-based screen of RNA-binding proteins (RBPs) regulating B cell activation and plasma cell (PC) differentiation, several eIF3 subunits were identified as potential regulators of these processes (Chen et al., 2025). To investigate the physiological roles of eIF3 subunits *in vivo*, we generated mice harboring conditional alleles for the *Eif3e* gene, which encodes a component of the eIF3 complex, and selectively deleted it in B cells. Strikingly, *Cγ1*^Cre^-mediated deletion of *Eif3e* led to spontaneous lymphocyte activation, lymphoid malignancy, and premature death.

## Results and discussions

### B cell–specific deletion of *Eif3e* led to lymphoma development

**Figure 1.**
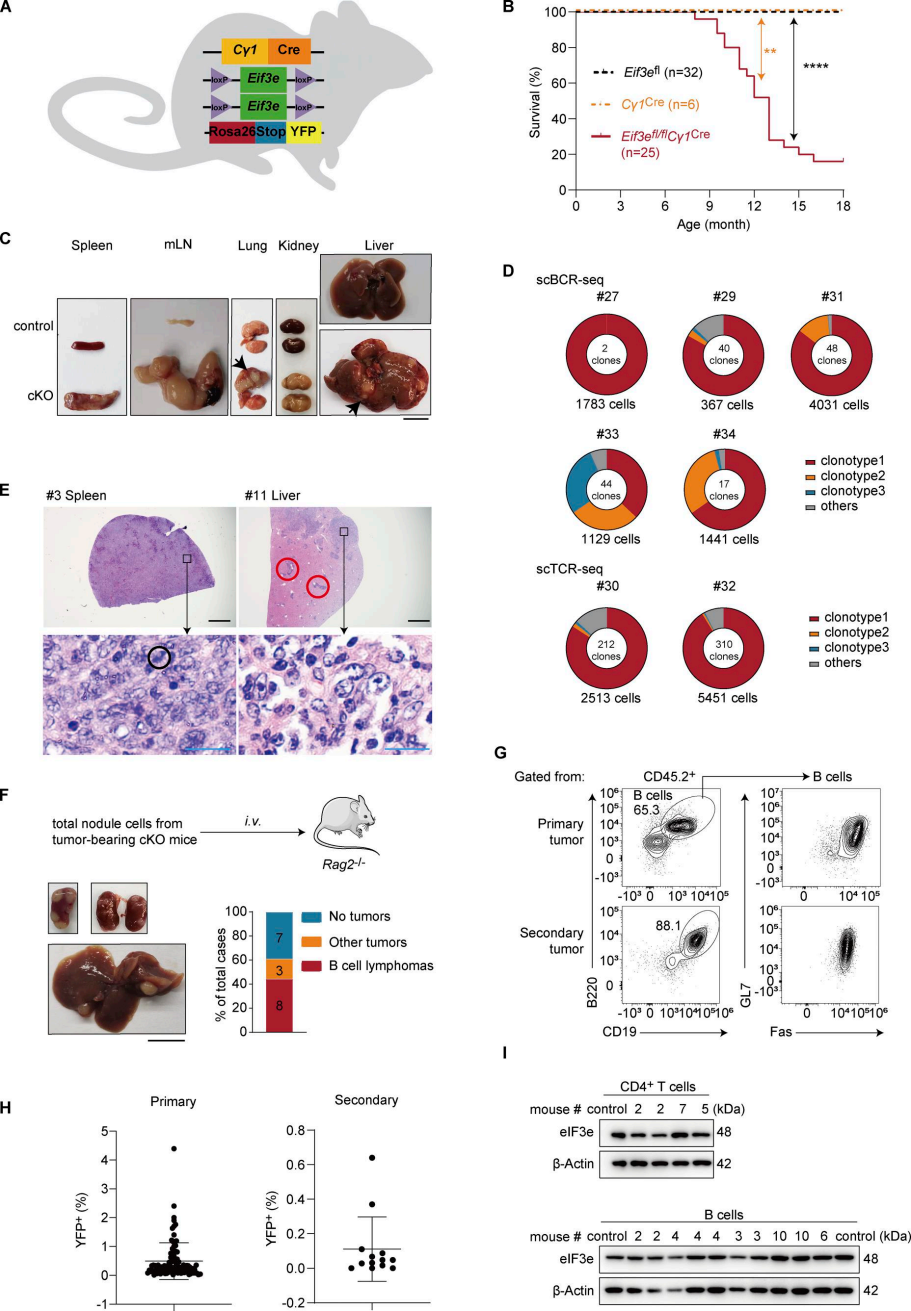
**Mice with B cell–specific deletion of *Eif3e* develop lymphoma. (A)** Schematic depiction of *Eif3e*-conditional knockout mice with YFP reporter (*Eif3e*^fl/fl^*Cγ1*^Cre^*Rosa26-YFP*^LSL^, termed cKO). **(B)** Kaplan–Meier survival curves of *Cγ1*^Cre^ (orange, *n* = 6), *Eif3e*^fl^ (black, *n* = 32), and cKO (red, *n* = 25) mice. **P < 0.01; ****P < 0.0001. Statistical significance is calculated using the log-rank test. **(C)** Splenomegaly, lymphadenopathy, and tumor cell infiltration (arrows) into the lung, kidney, and liver of 10- to 13-mo-old cKO mice. Scale bar, 1 cm. **(D)** Pie chart visualizing the distribution of the top three most enriched clonotypes in seven cKO mice. Six samples were primary tumors from mouse #29 (lung), #30 (liver), #31 (spleen), #32 (spleen), #33 (thymus), and #34 (lung and thymus). One sample was secondary tumor transplanted from primary nodule of mouse #27 (liver). Both the number of cells and clones are displayed. **(E)** Representative H&E staining of tumors in cKO mice. Top panels, low-power views (black scale bars, 1 mm). Bottom panels, high-power views (blue scale bars, 20 μm). **(F)** Tumor transplantation into *Rag2*^−/−^ mice. Upper panel, experimental outline. Lower left, representative photos of secondary tumors in recipient mice. Lower right, stacked bar plot summarizing the types of secondary tumors. Scale bar, 1 cm. **(G)** Flow cytometry analysis of primary and secondary B cell lymphoma in cKO and *Rag2*^−/−^ recipient mice, respectively. **(H)** Percentages of YFP^+^ cells in primary and secondary tumors. **(I)** Immunoblot analysis of eIF3e expression in purified CD4^+^ T and B cells from tumors in cKO mice. Controls were CD4^+^ T (upper panel) and B cells (lower panel) purified from the splenocytes of wild-type mice. H&E, hematoxylin and eosin. Source data are available for this figure: SourceData F1. The #numbers in the figure correspond to the mouse numbers in Table S1.

In our recent CRISPR/Cas9-based screen of RBPs regulating B cell activation and PC differentiation, several eIF3 subunits were identified as potential regulators of these processes (Chen et al., 2025). To investigate the physiological roles of eIF3 subunits *in vivo*, we generated mice harboring conditional alleles for *Eif3a*, *Eif3d*, and *Eif3e* and bred them with *Cγ1*^Cre^ mice, in which Cre recombinase is induced by transcription of the Ig γ1 constant region gene segment and deletes floxed alleles in activated B cells (Casola et al., 2006). *Rosa26-YFP*^LSL^ mice were crossed with those mice to track Cre activation *in vivo* (Srinivas et al., 2001). Colonies of the resulting mice were monitored over lifespan. Mutant mice deficient of *eIF3a* and *3d* (*Eif3a*^fl/fl^*Cγ1*^Cre^ and *Eif3d*^fl/fl^*Cγ1*^Cre^) appeared healthy and fertile over the period of observation (data not shown). Surprisingly, *Eif3e*^fl/fl^*Cγ1*^Cre^ mice started to die at the age of 8 mo. Among a cohort of 25 *Eif3e*^fl/fl^*Cγ1*^Cre^ mice, only 4 survived past 18 mo, while all control (*Eif3e*^fl/+^, *Eif3e*^fl/fl^, or *Cγ1*^Cre^) mice remained alive and healthy (Fig. 1, A and B). We therefore focused on the study of *Eif3e* hereafter.

Sick mice were sacrificed and macroscopically examined. *Eif3e*^fl/fl^*Cγ1*^Cre^ (termed cKO hereafter) mice exhibited splenomegaly and lymphadenopathy, often accompanied by multiple white nodules in the liver, lung, and thymus, and rarely in the kidney (Fig. 1 C and Table S1). Individual white nodules were excised and analyzed by flow cytometry. They were enriched with lymphocytes, mainly B and CD4^+^ T cells (Table S1). To analyze the clonality of lymphocytes from white nodules, we performed single-cell B cell receptor (BCR)/T cell receptor (TCR)-sequencing analysis of samples from seven cKO mice. All seven mice exhibited dominant B or T cell clonal expansion within the nodules (Fig. 1 D and Table S2), suggesting development of lymphomas.

Histopathological examination of diseased organs from seven cKO mice revealed common features of aggressive lymphoid neoplasia (Fig. 1 E and Table S3). Affected tissues showed dense infiltration by neoplastic lymphoid cells, often with marked effacement and distortion of normal tissue architecture. The infiltrates were variable in size and distribution and consisted of heterogeneous lymphoid populations, including small lymphocytes with condensed chromatin and larger transformed cells with open chromatin and prominent nucleoli. Mitotic figures were frequently observed, indicating high proliferative activity. Apoptotic debris and tingible body macrophages were also present in some cases, consistent with spontaneous cell death (Ito et al., 1999). In certain tissues, infiltrates were associated with vascular structures and extended to distort surrounding parenchyma. Together, these findings indicate that cKO mice develop highly infiltrative, histologically heterogeneous, and aggressive lymphoid tumors across multiple organs.

Mouse lymphoma cells differ from wild-type lymphocytes in their capacity to propagate disease upon transplantation into healthy mice. To determine whether cKO lymphoma cells are able to propagate disease, we transplanted total tumor cells from 18 tumor-bearing cKO mice into immunodeficient *Rag2*^−/−^ mice (Fig. 1 F). Among the 18 cases transplanted, 11 were able to establish secondary tumors in *Rag2*^−/−^ mice. Among secondary tumors, 8 were enriched with B cells that were mostly Fas^+^GL7^+^ (Table S4 and Fig. 1 G). Enrichment of B or T cells was not found in the other 3 cases. We termed those tumors enriched with non-B/non-T immune cells as other tumors. Moreover, when primary tumor cells from different involved organs of the same sick animal were transplanted into different *Rag2*^−/−^ mice, they grew into secondary tumors of the same phenotype (Table S1).

Surprisingly, all B and CD4^+^ T cells in primary and secondary tumors were negative for YFP and expressed normal amounts of eIF3e proteins (Fig. 1, H and I). Taken together, these findings suggest that eIF3e-sufficient lymphocytes unexpectedly underwent malignant transformation, developed into lymphomas, and caused premature death of cKO mice.

### *Eif3e* deficiency caused a lymphoproliferative disorder

**Figure 2.**
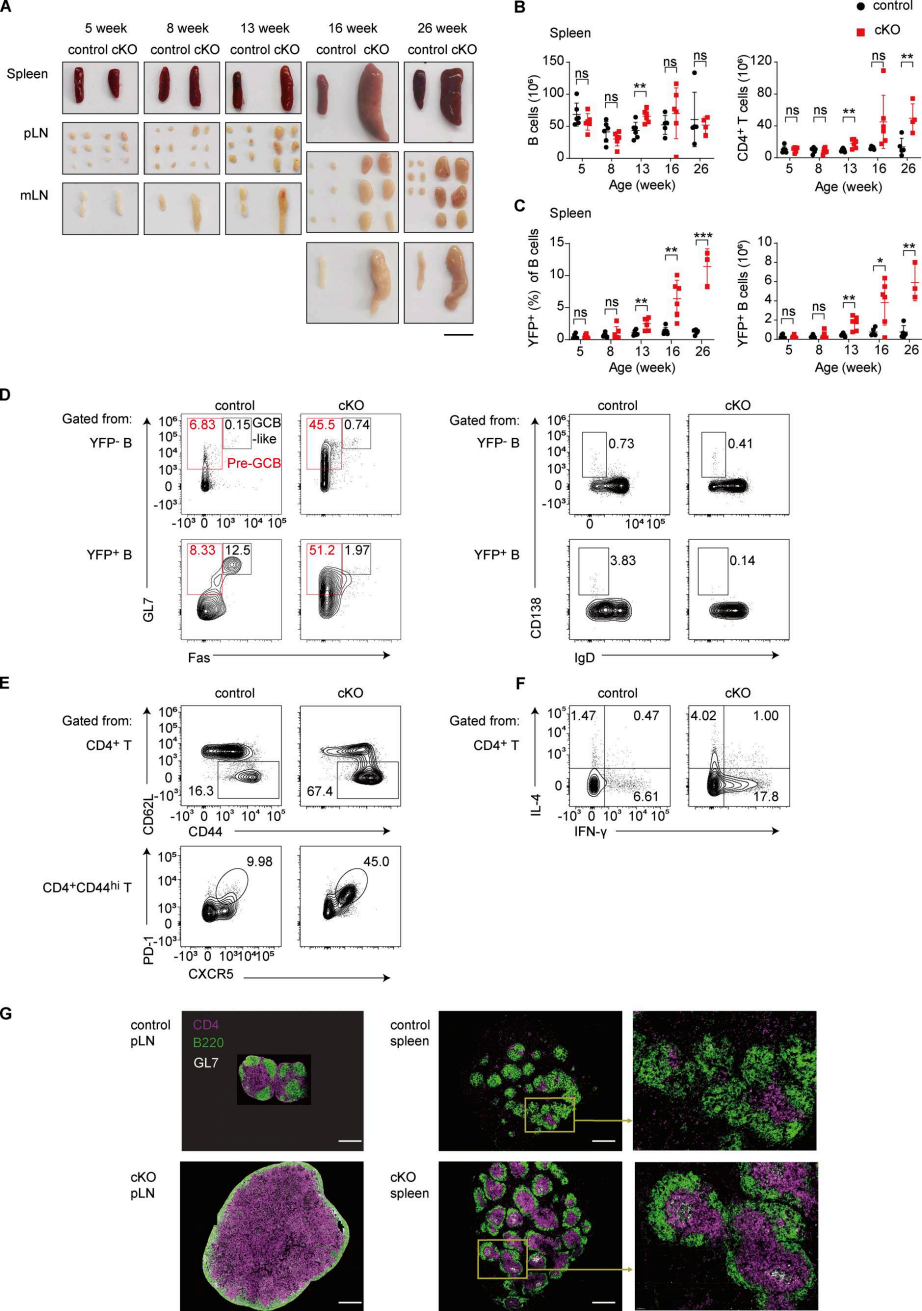
***Eif3e* deficiency causes lymphoproliferation. (A)** Representative photos of the spleens, pLNs, and mLNs from control (*Cγ1*^Cre^*Rosa26-YFP*^LSL^) and cKO (*Eif3e*^fl/fl^*Cγ1*^Cre^*Rosa26-YFP*^LSL^) mice at indicated ages. Scale bar, 1 cm. **(B and C)** Numbers of B and CD4^+^ T cells (B), and percentage and number of YFP^+^ B cells (C) in the spleen of control and cKO mice at indicated ages. Each symbol represents an individual mouse. Small horizontal lines indicate the mean (±SD). ns, not significant, P > 0.05; *P < 0.05; **P < 0.01; ***P < 0.001, determined by two-tailed unpaired Student’s *t* test. **(D–F)** Flow cytometry analysis of B and CD4^+^ T cells in the spleen of 16-wk-old control and cKO mice. **(G)** Immunofluorescence analysis of pLN and spleen from 16-wk-old control and cKO mice. Scale bars, 500 μm. Two (A–F) and three (G) independent experiments were performed. pLNs, peripheral lymph nodes; mLNs, mesenteric lymph nodes.

**Figure S1.**
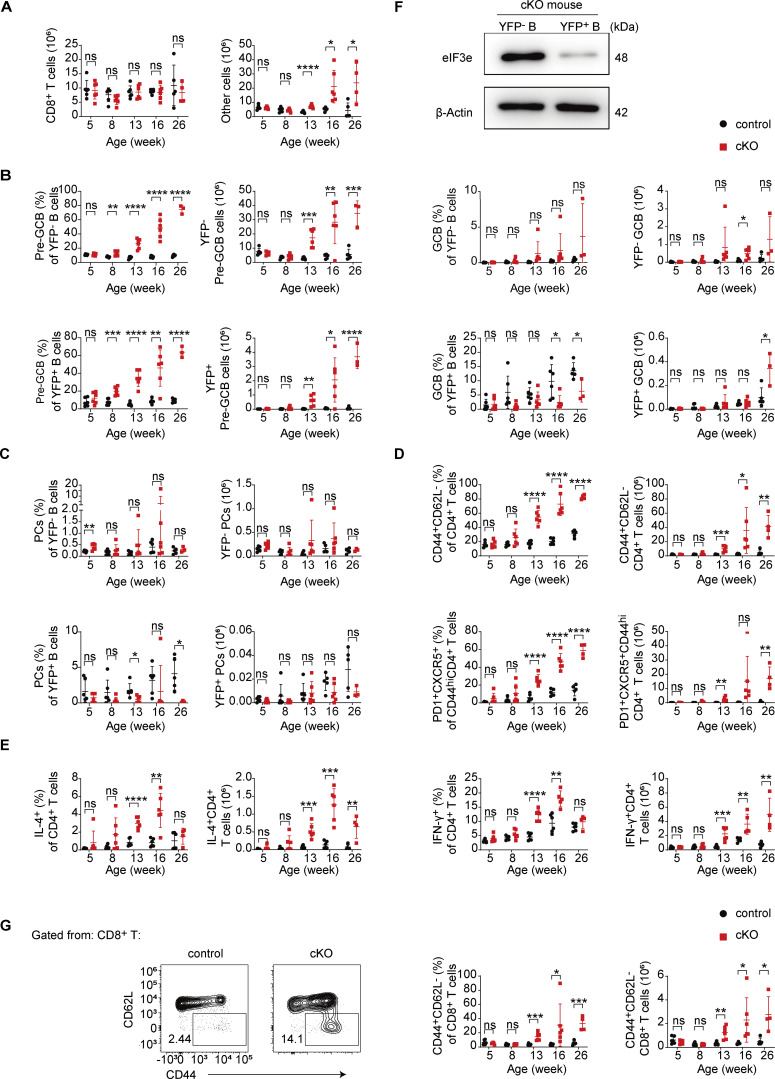
**Percentage, number, and activation status of T and B cells in cKO mice. (A–E)** Percentage and number of T, B, and other immune cell subsets in the spleen of control and cKO mice at indicated ages. **(F)** Immunoblot analysis of eIF3e expression in YFP^−^ and YFP^+^ B cells from 8-mo-old cKO mice. **(G)** Flow cytometry analysis of CD8^+^ T cells in the spleen of 16-wk-old control and cKO mice. Left: Representative FACS plots. Right: Number and percentage of indicated T cell populations. Each symbol represents an individual mouse. Small horizontal lines indicate the mean (±SD). ns, not significant, P > 0.05; *P < 0.05; **P < 0.01; ***P < 0.001; ****P < 0.0001, determined by two-tailed unpaired Student’s *t* test. Two independent experiments (A–G) were performed. Source data are available for this figure: SourceData FS1.

We then analyzed cKO mice at different ages to investigate how *Eif3e* deletion in activated B cells led to malignant transformation of eIF3e-sufficient lymphocytes. At young ages, cKO mice were healthy and fertile. cKO mice exhibited slightly enlarged spleen and lymph nodes at 8 wk, and obvious splenomegaly and lymphadenopathy were observed at 16 wk or older (Fig. 2 A). The numbers of CD4^+^ and other immune cells in the spleen started to increase at 12 wk, while the numbers of splenic B cells and CD8^+^ T cells remained similar to age-matched control mice (Fig. 2 B and Fig. S1 A).

Consistent with previous reports (Srinivas et al., 2001), *Cγ1*^Cre^-mediated deletion occurred only in a small number of B cells in the spleen of young cKO and control mice (Fig. 2 C). Immunoblot analysis of YFP^+^ B cells from cKO mice confirmed deletion of *Eif3e* in these cells (Fig. S1 F). Notably, the percentage and number of YFP^+^ B cells increased over time in cKO mice, indicating *Eif3e*-deficient B cells gained a competitive advantage or more naïve B cells became activated and turned on *Cγ1*^Cre^ expression (Fig. 2 C). In 16-wk-old or older cKO mice, both YFP^+^ and YFP^−^ B cells showed elevated expression of GL7, a marker for B cell activation (Cervenak et al., 2001). While YFP^+^ B cells differentiated into GL7^+^Fas^+^ GCB-like and CD138^+^ PCs in control mice, YFP^+^ B cells in cKO mice remained GL7^+^, with few of them upregulating Fas expression, suggesting that they were retained at the pre-GCB stage (GL7^+^Fas^−^) (Fig. 2 D; and Fig. S1, B and C). CD4^+^ T cells underwent significant expansion in cKO mice, accompanied by enhanced activation and differentiation into PD-1^+^CXCR5^+^ T_FH_-like cells, as well as increased production of IL-4 and IFN-*γ* (Fig. 2, B, E, and F; and Fig. S1, D and E). CD8^+^ T cells were not expanded in number but showed moderately increased activation in cKO mice (Fig. S1, A and G).

Immunofluorescence staining of the spleen and lymph nodes of 16-wk-old cKO mice showed drastic expansion of CD4^+^ T cell zones, pushing B cells to the periphery of lymphoid follicles. GL7^+^ cells were present in the CD4^+^ T cell zones of the spleen and at the T–B borders in lymph nodes (Fig. 2 G). Different from the disrupted follicular structures and random mixtures of B and T cells often observed in autoimmune mouse models (Xiao et al., 2008), the T–B borders remained clearly demarcated in cKO mice (Fig. 2 G). Together, these results demonstrate that *Cγ1*^Cre^-mediated deletion of *Eif3e* in activated B cells led to systemic activation of both B and T cells, resulting in a lymphoproliferative disorder at a relatively young age.

### A feedforward loop of T–B interaction drives lymphoproliferation in *Eif3e*-deficient mice

**Figure 3.**
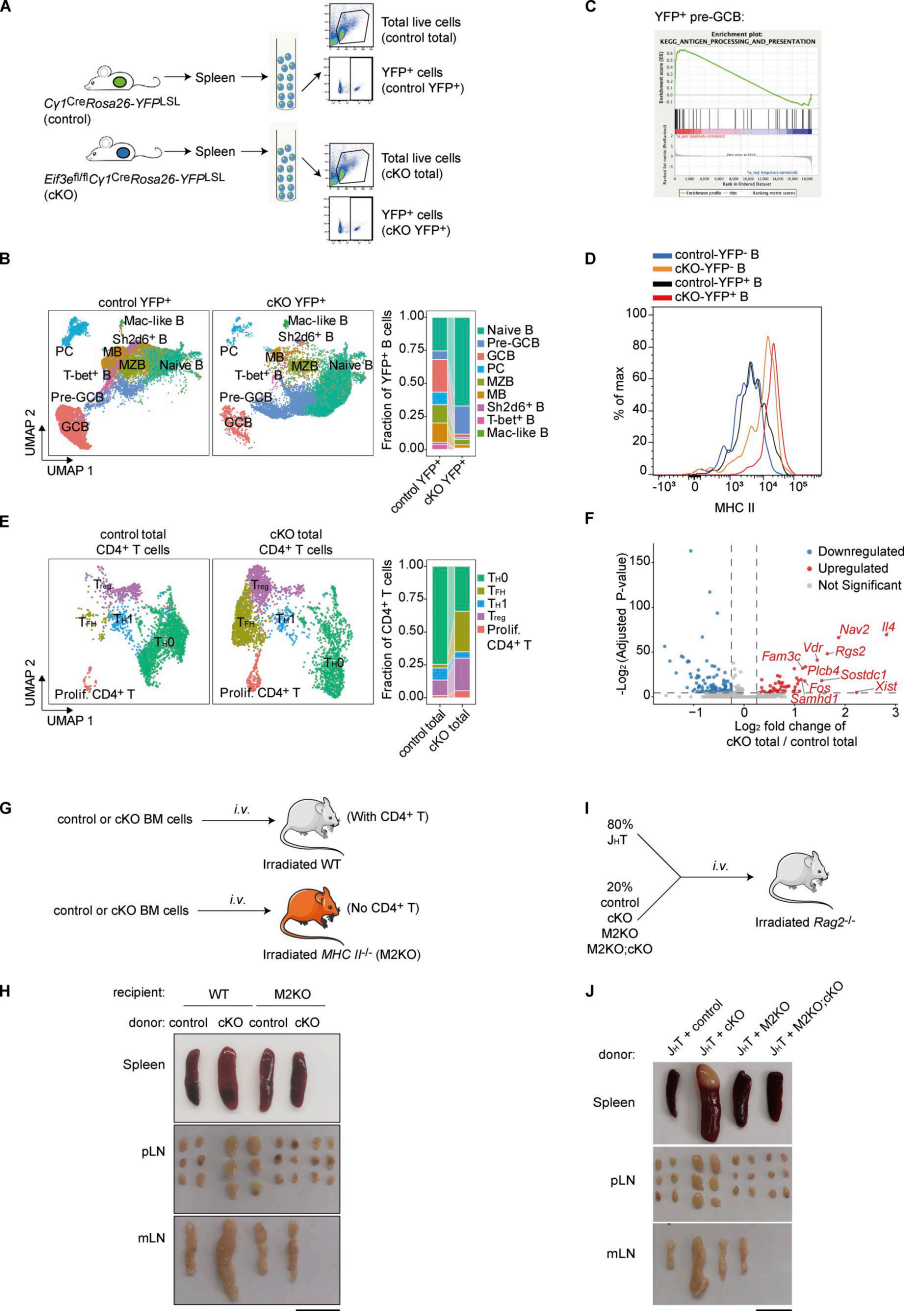
***Eif3e* deficiency initiates a feedforward loop of T–B interaction and lymphocyte activation. (A)** Experimental outline for scRNA-seq analysis of total and YFP^+^ cells in the spleen of 2-mo-old control and cKO mice. **(B)** UMAP clustering analysis of B cell subpopulations in control and cKO YFP^+^ samples. A stacked bar plot summarizes fractions of each B cell subpopulation. **(C)** GSEA of top DEGs in cKO vs. control YFP^+^ pre-GCB cells. **(D)** Flow cytometry histogram showing MHC II intensity on YFP^−^ and YFP^+^ B cells from 12-wk-old control and cKO mice. **(E)** UMAP clustering analysis of subpopulations of CD4^+^ T cells in control and cKO total samples. A stacked bar plot summarizes fractions of each CD4^+^ T cell subpopulation. **(F)** Volcano plot showing DEGs in the T_FH_ cell subpopulations of cKO versus control total cells. The cutoff was set at P <0.05 and absolute log2FC >0.25. **(G–J)** Functional analysis of CD4^+^ T cells and B cell expression of MHC II in the development of lymphoproliferation disorder in cKO mice. **(G and I)** Experimental outline for generation of bone marrow chimeras without CD4^+^ T cells (G) or B cell–specific deletion of MHC II (I). **(H and J)** Representative photos of spleens, pLNs, and mLNs from indicated chimeras at 3 mo after reconstitution. Scale bars, 1 cm. scRNA-seq data (B, C, E, and F) from two independent biological replicates were merged for analysis. Data (D, H, and J) are representative of two independent experiments. pLNs, peripheral lymph nodes; mLNs, mesenteric lymph nodes.

**Figure S2.**
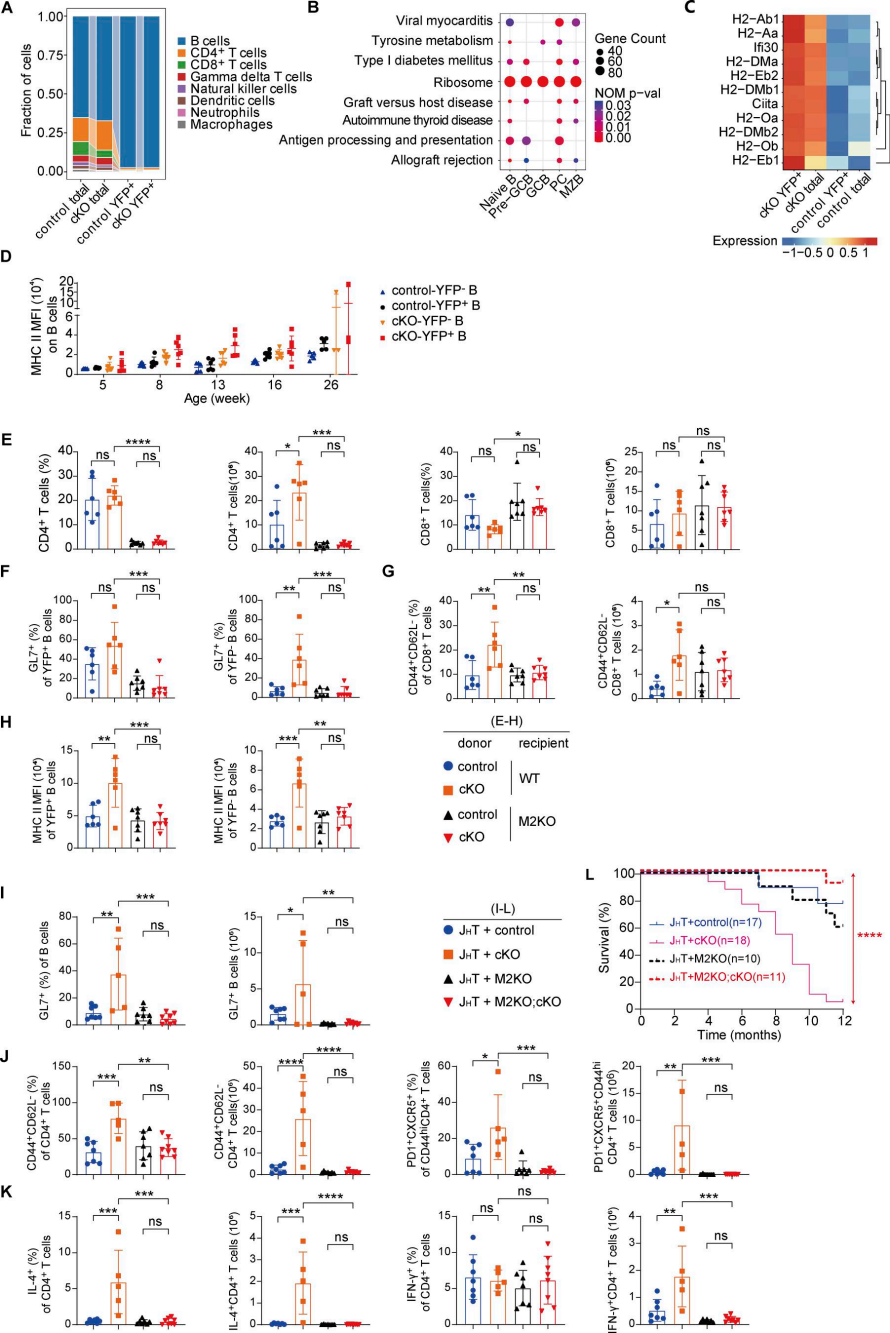
**scRNA-seq and functional analyses reveal that the cKO phenotype relies on CD4** ^**+**^ **T cell interactions via MHC II. (A)** Stacked bar plot summarizes fractions of main cell populations across four experimental groups: control total, cKO total, control YFP^+^, and cKO YFP^+^ samples in Fig. 3 A. **(B)** Bubble plot showing the top-ranked KEGG enriched terms among upregulated genes in main B cell subpopulations, based on GSEA comparing cKO and control YFP^+^ cells. The bubble size represents gene count of the enriched gene set, and color indicates the normalized P value. **(C)** Heatmap plot depicting RNA expression of the top-ranked DEGs in antigen processing and presentation pathway in cKO and control pre-GCB cells. The RNA expression level of each gene is normalized. **(D)** Statistical analysis of mean fluorescence intensity of MHC II expression on various B cell populations in control and cKO mice at indicated ages. **(E–H)** Flow cytometry analysis of splenocytes (E), B cells (F), CD8^+^ T cells (G), and MHC II expression on YFP^+^ and YFP^−^ B cells (H) in the spleen of bone marrow chimeras in Fig. 3 G. Shown are bar graphs summarizing percentages and numbers of indicated cell populations. **(I–L)** Flow cytometry analysis of B (I) and CD4^+^ T cells (J and K) in the spleen of bone marrow chimeras in Fig. 3 I. Shown are bar graphs summarizing percentages and numbers of indicated cell populations. **(L)** Kaplan–Meier survival curves of bone marrow chimeras in Fig. 3 I. Each symbol represents an individual mouse. Small horizontal lines indicate the mean (±SD). Statistical significance is calculated using either one-way ANOVA followed by Tukey’s multiple-comparisons test (E–K) or the log-rank test (L). ns, not significant, P > 0.05; *P < 0.05; **P < 0.01; ***P < 0.001; ****P < 0.0001. scRNA-seq data (A–C) from two independent biological replicates were merged for analysis. Two independent experiments (D–L) were performed.

To gain insights into the cellular mechanisms underlying the lymphoproliferative disorder developed in cKO mice, we performed single-cell RNA-sequencing (scRNA-seq) analysis of YFP^+^ and total splenocytes from cKO and control mice at the age of 2 mo (Fig. 3 A), when lymphocyte activation and proliferation had not yet become obvious (Fig. 2, A and B; and Fig. S1). After pooling two biological replicates of scRNA-seq data, the following numbers of cells were obtained for analysis: control total (21,127), cKO total (21,058), control YFP^+^ (20,928), and cKO YFP^+^ (20,595). The major lymphocyte populations were clustered, and their fractions in each sample were calculated (Fig. S2 A). There were no obvious differences between total cells from control and cKO mice (termed control total and cKO total, respectively). YFP^+^ cells were further clustered into different B cell subsets. An increase in the fractions of naïve B and pre-GC cells was observed among cKO YFP^+^ cells, accompanied by a decrease in the fraction of GCB cells and near absence of plasma cells (PCs, Fig. 3 B), suggesting impaired differentiation of *Eif3e*-deficient B cells at the pre-GCB-to-GCB transition, which exacerbated at the GCB-to-PC transition. This is consistent with previous flow cytometry and immunofluorescence analyses of the spleen of 16-wk-old cKO mice (Fig. 2, D and G). We performed gene set enrichment analysis (GSEA) of differentially expressed genes (DEGs) in YFP^+^ B cell subpopulations utilizing KEGG pathways. Interestingly, “antigen processing and presentation” was exclusively enriched in naïve B, pre-GCB, and PCs among YFP^+^ cKO cells (Fig. 3 C and Fig. S2 B). Among the top-ranked DEGs were genes of the MHC II pathway (Fig. S2 C). Elevated surface expression of MHC II on YFP^+^ B cells of cKO mice was confirmed by flow cytometry (Fig. 3 D and Fig. S2 D).

Previous studies have shown that elevated MHC II expression on B cells promotes antigen presentation to and cognate interaction with CD4^+^ T cells. We speculated that *Eif3e*-deficient B cells gained a competitive advantage in interacting with CD4^+^ T cells and directing their differentiation into T_FH_ cells (Schwickert et al., 2011; Yeh et al., 2022). Indeed, a drastic increase in the fraction of T_FH_ cells was observed among CD4^+^ T cells from cKO mice (Fig. 3 E), consistent with previous flow cytometry analysis (Fig. 2 E). Further analysis of transcriptomes of T_FH_ cells from control and cKO mice revealed a significant increase in IL-4, but not IFN-γ, mRNA levels in T_FH_ cells from cKO mice (Fig. 3 F and Table S5). This suggests that YFP^+^ B cells initially direct CD4^+^ T cells toward an IL-4–producing phenotype, while the high expression of IFN-γ observed in cKO mice after 12 wk of age could be a consequence of immune dysregulation (Hodge et al., 2014). IL-4 is a cytokine with pleiotropic activity in the immune system. It promotes B cell activation, proliferation, survival, and class switch to IgG1 and IgE, and upregulates MHC II and CD23 expression (Erb et al., 1994; Erb et al., 1997; Granato et al., 2014). Elevated IL-4 expression by CD4^+^ T cells may cause spontaneous activation of and aberrant MHC II upregulation on YFP^−^ B cells in cKO mice (Fig. 2 D; Fig. 3 D; Fig. S1, B and C; and Fig. S2 C). We speculate that *Eif3e*-deficient B cells promote cognate T–B interactions, leading to IL-4 secretion by those T_FH_-like cells and activation of bystander B cells, which further induce *Cγ1*^*Cre*^ expression and trigger additional rounds of *Eif3e* deletion. This creates a self-sustaining feedforward loop of aberrant activation of *Eif3e*-sufficient B and T cells that ultimately culminates in a lymphoproliferative disorder and, in some cases, the development of lymphoma.

To investigate the role of CD4^+^ T cells in this feedforward loop of lymphocyte activation in cKO mice, we generated mixed bone marrow chimeras by transferring control or cKO bone marrow cells into irradiated wild-type or *MHC II*^−/−^ mice (termed M2KO hereafter, Fig. 3 G). As MHC II expression in the thymus is essential for the development of CD4^+^ T cells (Grusby et al., 1991), all M2KO recipients failed to generate this T cell subset after bone marrow reconstitution (Fig. S2 E). Microscopic examination of lymphoid organs from chimeras 3 mo after reconstitution showed that all wild-type recipients reconstituted with cKO bone marrow cells developed splenomegaly and lymphadenopathy, whereas none of the M2KO recipients did (Fig. 3 H). A large population of GL7^+^ B cells were present in the former group, but absent in the latter (Fig. S2 F). Enhanced activation of CD8^+^ T cells was also absent in M2KO chimeras (Fig. S2 G). Notably, MHC II expression on YFP^+^ and YFP^−^ B cells was brought back to normal levels in the M2KO chimeras reconstituted with cKO bone marrow cells, demonstrating a critical role of CD4^+^ T cells in mediating MHC II upregulation on cKO B cells (Fig. S2 H).

We next assessed the role of MHC II expression on *Eif3e*-deficient B cells in the development of the lymphoproliferative disorder in cKO mice. Toward this goal, we bred cKO with M2KO mice (termed M2KO;cKO hereafter) and then generated bone marrow chimeras by reconstituting *Rag2*^*−/−*^ mice with a mix of bone marrow cells from J_H_T (80%) and control, cKO, M2KO, or M2KO;cKO mice (20%) (Fig. 3 I). J_H_T mice fail to produce functional B cells (Gu et al., 1993). Therefore, all B cells in the resulting chimeras originated from the other donor bone marrow cells, whereas the majority of other immune cells originated from J_H_T mice and were wild-type. Accordingly, MHC II deficiency was restricted exclusively to B cells in chimeras reconstituted with J_H_T and M2KO or M2KO;cKO bone marrow cells. Splenomegaly and lymphadenopathy were observed in chimeras reconstituted with J_H_T and cKO bone marrow cells, but not in any other chimeras (Fig. 3 J). The accumulation of GL7^+^ B cells and T_FH_-like CD4^+^ T cells, as well as excessive production of IL-4, was absent in the chimeras reconstituted with J_H_T and M2KO;cKO bone marrow cells (Fig. S2, I–K). Importantly, the absence of MHC II expression on B cells also prevented premature death of chimeras reconstituted with J_H_T and M2KO;cKO bone marrow cells (Fig. S2 L). Taken together, these results demonstrate the critical importance of B cell expression of MHC II and CD4^+^ T cells in the development of lymphoproliferative diseases in cKO mice.

### eIF3e represses costimulatory molecule expression

**Figure 4.**
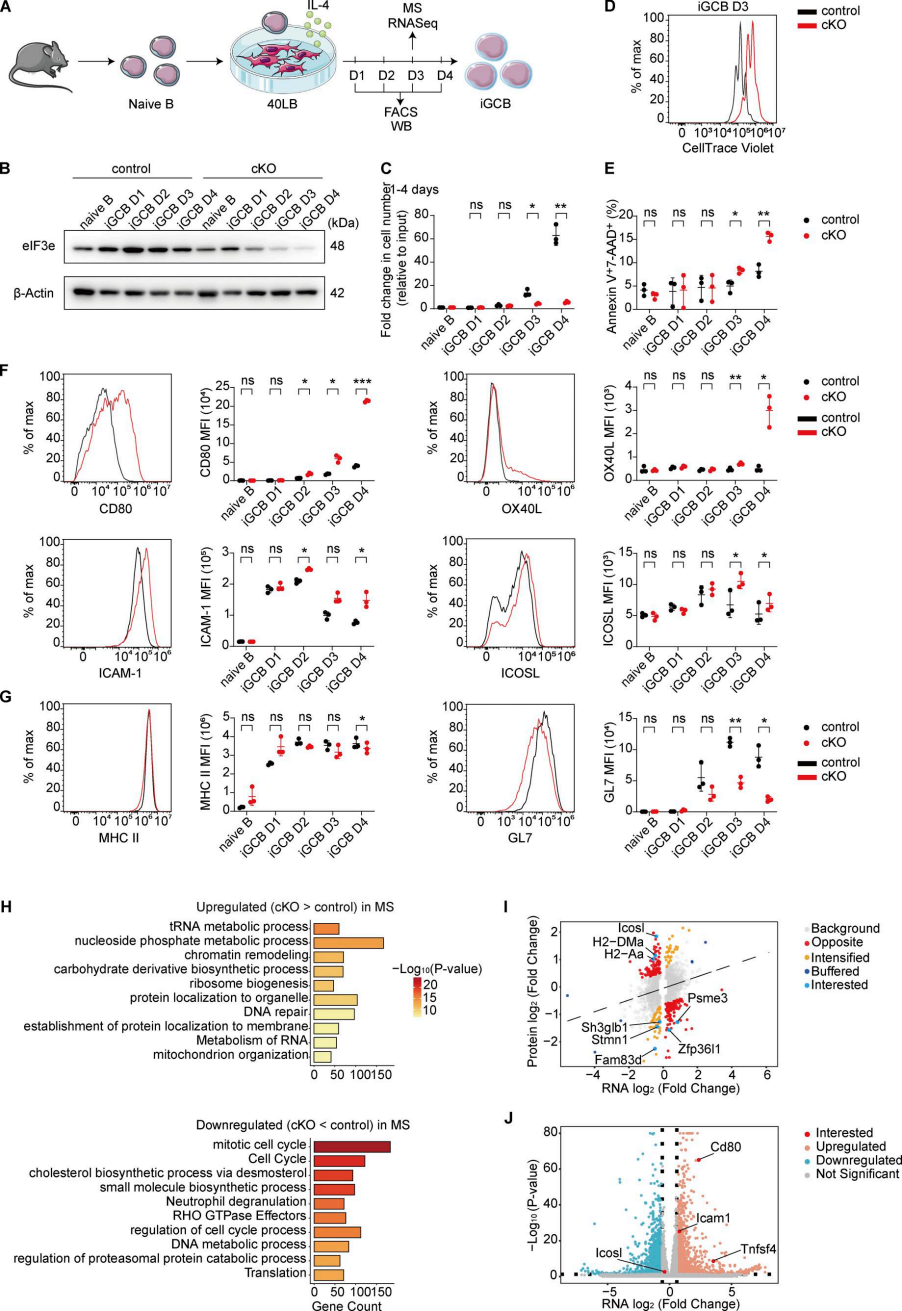
**eIF3e represses expression of costimulatory and adhesion molecules in B cells. (A)** Experimental outline for B cell *in vitro* activation assay. The *in vitro* activated B cells were sorted for RNA-seq, WB, and flow cytometry analysis (FACS). **(B–E)** Analysis of eIF3e expression (B), cell proliferation (C), and death (E) at indicated time points. Cell proliferation was calculated by dividing the number of activated cells by the number of input cells. **(D)** CellTrace Violet intensity assay of cell proliferation. Naïve B cells were labeled with CellTrace Violet, cultured in the *in vitro* activation system in A for 3 days, and analyzed by flow cytometry for CellTrace Violet intensity. **(F and G)** Flow cytometry analysis of cell surface proteins at indicated time points. Representative FACS histograms (also shown in Fig. S3 D, column 4) and statistical analyses of MFI are shown. **(H)** Metascape analysis of genes upregulated (top) and downregulated (bottom) at the protein level in eIF3e-deficient day 3 iGCBs relative to control, as determined by LC-MS/MS (Q <0.05 and |AVG.Log_2_.Ratio| >0.3). **(I)** Scatter plots showing log2FCs in protein and RNA expression levels in eIF3e-deficient B cells relative to control at iGCB day 3. The 343 highlighted genes exhibit altered translation efficiency, with interested targets marked by gene names. These 343 translationally regulated genes were classified into distinct functional categories: Opposite (RNA and protein abundance changed in biologically opposing directions), Intensified (protein FC exceeded RNA log2(FC) by >0.5 log_2_ units), and Buffered (log_2_(FC) in protein response was blunted by >0.5 compared with the RNA shift). **(J)** Volcano plot showing DEGs (|FC| >1.5, adjusted P <0.05) identified by RNA-seq in cKO versus control B cells cultured in the *in vitro* activation system in A for 3 days. Statistical analysis of cell proliferation (C), cell death (E), and MFI (F and G) was performed using paired two-tailed Student’s *t* test for the control and cKO CD19^+^ iGCBs. ns, not significant, P > 0.05; *P < 0.05; **P < 0.01; ***P < 0.001. Each dot represents one mouse. Three independent experiments (B–G) or three biological replicates (H–J) were performed. WB, western blot; MFI, mean fluorescence intensity. Source data are available for this figure: SourceData F4.

**Figure S3.**
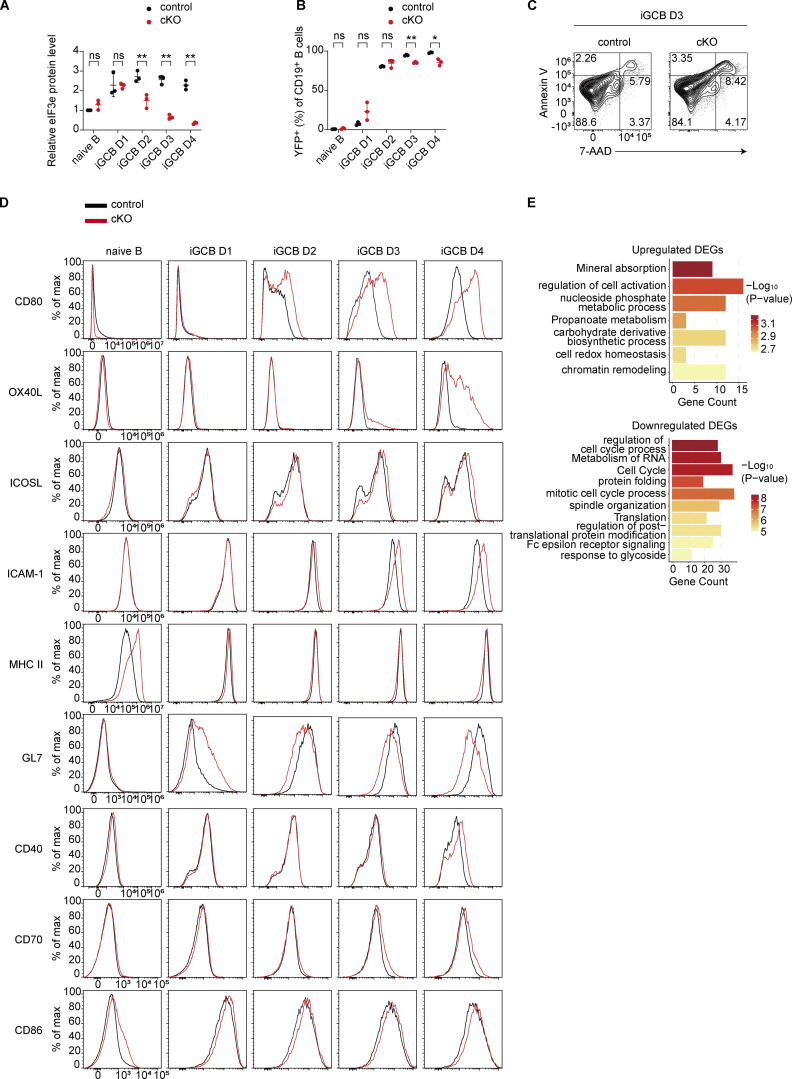
**Analysis of cKO B cells upon *in vitro* activation. (A–D)** Statistical analyses of the eIF3e protein level (A), percentage of YFP^+^ cells (B), FACS analysis of cell death (C), and cell surface protein expression (D) in control and cKO B cells at indicated time points of *in vitro* activation (Fig. 4 A). The representative FACS histograms in panel D (column 4) for the iGCB day 3 time point are also shown in Fig. 4, F and G. Each symbol in A and B represents an individual mouse. Small horizontal lines indicate the mean (±SD). Statistical significance is calculated using paired two-tailed Student’s *t* test (A and B). ns, not significant, P > 0.05; *P < 0.05; **P < 0.01. Three independent experiments (A–D) were performed. **(E)** Metascape analysis of genes highlighted in Fig. 4 I with upregulated (top) and downregulated (bottom) translation efficiency. Source data are available for this figure: SourceData FS3.

We next utilized an *in vitro* culture system of B cell activation and differentiation to elucidate the cellular and molecular mechanisms underlying eIF3e functions. Naïve B cells from control and cKO mice were cocultured with 40LB cells (BALB/c 3T3 cells stably expressing CD40L and BAFF). In the presence of IL-4, naïve B cells were activated, underwent extensive proliferation, and differentiated into *in vitro* germinal center B cells (iGCBs) (GL7^+^Fas^+^Bcl6^+^) (Fig. 4 A) (Nojima et al., 2011). As shown in Fig. 4 B and Fig. S3 A, *Cγ1*^Cre^-mediated deletion initiated a decrease in eIF3e protein levels in cKO B cells at day 2 and the depletion became obvious at days 3 and 4. YFP reporter expression also indicated an increase in Cre activity over time in this system (Fig. S3 B), suggesting the effect caused by eIF3e depletion occurred primarily at days 3 and 4. We then performed time course experiments to compare cell proliferation and death of control and cKO B cells. While control B cells expanded drastically at days 3 and 4, cKO B cells showed only slight expansion (Fig. 4 C), likely due to impaired proliferation and increased death (Fig. 4, D and E; and Fig. S3 C). These results demonstrated that eIF3e is required for B cell survival and proliferation.

It is well known that costimulatory and adhesion molecules play important roles in promoting T–B interactions. We investigated the expression of costimulatory and adhesion molecules on cKO B cells. As shown in Fig. 4 F and Fig. S3 D, cKO B cells exhibited much elevated expression of costimulatory molecules CD80, OX40L, and, to a lesser degree, ICOSL (Du et al., 2024; Good-Jacobson et al., 2012; Liu et al., 2015; O'Neill et al., 2007). ICAM-1, an adhesion molecule that promotes B cell interactions with T_FH_ cells, was also upregulated in cKO B cells (Zaretsky et al., 2017). Importantly, upregulation of MHC II and GL7 was not observed in these cKO B cells (Fig. 4 G), suggesting that their upregulation *in vivo* was an indirect consequence of B cell activation. Indeed, cKO B cells did not exhibit upregulation of MHC II and GL7 in the bone marrow chimeras without CD4^+^ T cells (Fig. S2, F and H).

Day 3 control and cKO iGCBs were further analyzed by RNA-seq and LC-MS/MS. We first examined global proteomic changes in cKO cells. Upregulated proteins were enriched in pathways related to RNA metabolism and ribosome biogenesis, macromolecule metabolism, chromatin remodeling and DNA repair, and protein localization and membrane transport (Fig. 4 H and Table S6). Downregulated proteins were enriched in pathways such as cell cycle and lipid biosynthesis, consistent with the impaired proliferation of cKO B cells (Fig. 4 H and Table S6) (Zhu and Thompson, 2019). To assess the contribution of translational control, we performed an integrative analysis of matched RNA-seq and quantitative proteomics datasets, focusing on DEGs identified in both datasets. To distinguish translational regulation from transcriptional changes, we generated a linear regression model (Protein log_2_ fold change [FC] ∼ RNA log_2_FC). Genes with standardized residuals (Z-score) >1.2 were defined as translationally regulated. This analysis identified 343 genes with altered translation efficiency, which were subsequently subjected to GO analysis (Fig. S3 E and Table S7). Genes with reduced translation efficiency were enriched in pathways related to regulation of cell cycle process, suggesting eIF3e controls cell cycle primarily at the translational level. Among genes with increased translation efficiency, pathways involved in regulation of cell activation were enriched, including MHC class II pathway genes such as *H2-Aa* and *H2-Dma* (Fig. 4 I). Among the costimulatory and adhesion molecules differentially expressed in cKO B cells (Fig. 4 F), *Cd80*, *Tnfsf4* (encoding OX40L), and *Icam1* were upregulated at the mRNA level, whereas *Icosl* showed increased translation efficiency *(*Fig. 4, I and J; and Table S8). Together, these results suggest that eIF3e regulates gene expression through multiple mechanisms to maintain appropriate expression levels to preserve immune homeostasis.

### CD80-mediated B–T interactions initiate a feedforward loop of lymphocyte activation

**Figure 5.**
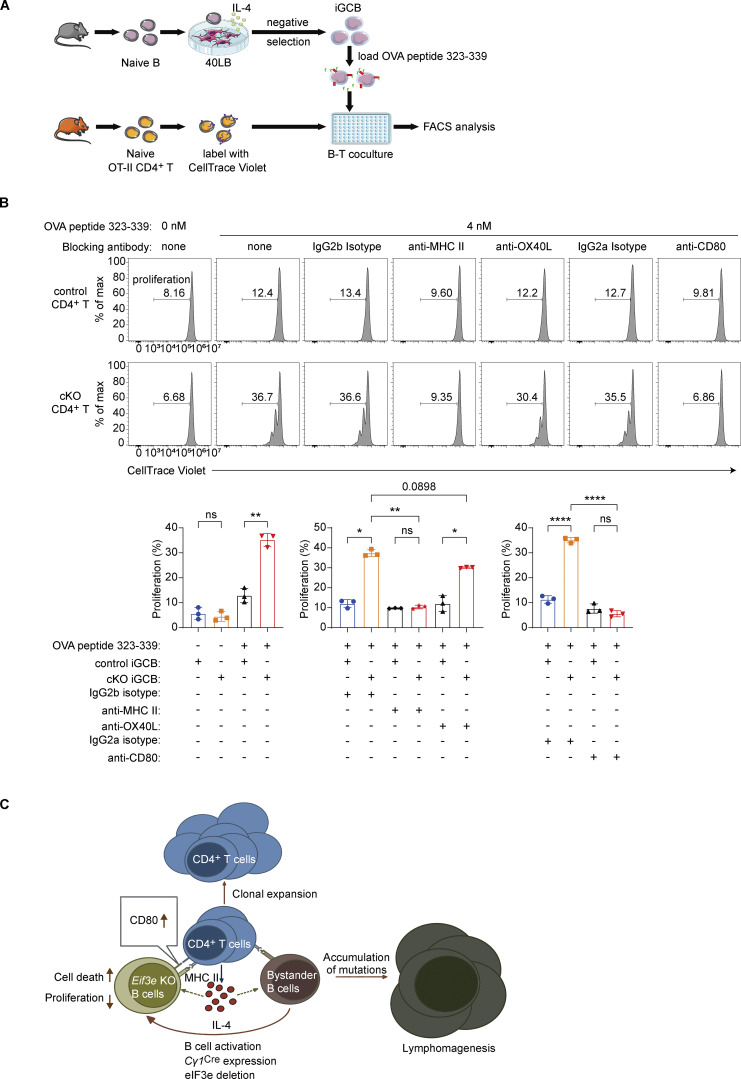
**CD80 upregulation by *Eif3e*-deficient B cells drives antigen-dependent CD4** ^**+**^ **T cell activation. (A)** Experimental outline for B–T cell coculture. Naïve OT-II CD4^+^ T cells labeled with CellTrace Violet were cocultured with OVA peptide 323–339–loaded iGCBs for 2.5 days. Proliferation of OT-II CD4^+^ T cells was measured by flow cytometry. **(B)** Upper panels: Representative FACS plots showing proliferation of OT-II CD4^+^ T cells cocultured with cKO or control iGCBs under indicated conditions. Below: Bar graphs summarizing proliferation of CD4^+^ T cells. Statistical analysis was performed using paired two-tailed Student’s *t* test. ns, not significant, P > 0.05; *P < 0.05; **P < 0.01; ****P < 0.0001. Three independent experiments (B) were performed. **(C)** Graphical abstract. *Eif3e* deficiency in B cells leads to elevated expression of CD80, a feedforward loop of T–B interaction, and aberrant activation and proliferation of B and CD4^+^ T cells, which culminate in malignant transformation of eIF3e-sufficient B or T cells.

To investigate the roles of these costimulatory and adhesion molecules, we established an *in vitro* coculture system using iGCBs and naïve OT-II CD4^+^ T cells (Fig. 5 A) (Guberman Bracha et al., 2025; Liang et al., 2018). When control or cKO iGCBs were cocultured with naïve OT-II CD4^+^ T cells in the absence of antigen, neither population induced T cell proliferation (Fig. 5 B). Upon addition of a low dose (4 nM) of OVA peptide 323–339, cKO iGCBs, but not control iGCBs, promoted robust OT-II CD4^+^ T cell proliferation. Notably, MHC II blockade completely abrogated the response, confirming that CD4^+^ T cell activation by cKO B cells is strictly dependent on MHC II–mediated antigen presentation. Similarly, CD80 blockade completely abrogated this response, while OX40L blockade produced only a modest reduction (Fig. 5 B). These findings indicate that CD80 upregulation on cKO iGCBs is the primary driver of CD4^+^ T cell activation and that this B–T interaction is antigen-dependent (Tanaka et al., 2020). We propose that *Eif3e*-deficient B cells upregulate costimulatory and adhesion molecules, enabling presentation of self-antigens to cognate CD4^+^ T cells and thereby promoting their activation and differentiation into IL-4–secreting T_FH_-like cells. These cells, in turn, activate bystander B cells, upregulate MHC II expression, induce *Cγ1*^Cre^ expression, and trigger additional rounds of *Eif3e* deletion, whereas *Eif3e*-deficient B cells undergo premature death. This process establishes a self-sustaining feedforward loop of lymphocyte activation that culminates in lymphoproliferative disease. Sustained activation and expansion of B and T cells may further promote the accumulation of genetic lesions in a subset of cells, ultimately driving malignant transformation of *Eif3e*-sufficient lymphocytes and lymphoma development (Fig. 5 C).

This study sheds some light on the long-standing paradox that eIF3e can function as both an oncogene and a tumor suppressor. Clinically, eIF3e expression is elevated in glioblastoma (Sesen et al., 2014) and colon cancer (Li et al., 2014), where it promotes global translation and cellular proliferation, whereas its expression is reduced in non–small-cell lung carcinoma (Buttitta et al., 2005) and certain breast cancers (Marchetti et al., 2001), in which its loss facilitates metastasis through factors such as HIF2α (Gotoh-Saito et al., 2022). Our findings offer a plausible explanation to the latter context: loss or reduced expression of eIF3e in one cell population may orchestrate a protumorigenic inflammatory microenvironment that promotes tumor development in genetically intact neighboring cells. The aberrant T_FH_ expansion and malignant transformation of bystander B cells observed in our model resemble features of angioimmunoblastic T cell lymphoma, in which mutant T_FH_ cells establish an inflammatory milieu that recruits and ultimately transforms bystander B cells into secondary lymphomas (Leca et al., 2023). Although the initiating event in our model arises in B cells rather than T cells, the resulting chronic inflammatory niche similarly drives the transformation of bystander lymphocytes.

To our knowledge, this is the first *in vivo* demonstration that a specific translation initiation factor safeguards against lymphoproliferative disease. Although the precise mechanisms by which eIF3e selectively regulates individual mRNA transcripts remain to be elucidated, our findings highlight translational control as a direct regulator of immune homeostasis. Future investigations are warranted to identify additional translation factors and their mechanism of action in preserving immune tolerance and preventing lymphomagenesis.

## Materials and methods

### Mice

All mice were maintained on the C57BL/6J genetic background. *Eif3e*^fl/fl^ mice were generated by Dr. Dieter A. Wolf (Westlake University, Hangzhou, China) (Lin et al., 2020). *MHC II*^−/−^ (Jax stock 003374) (Madsen et al., 1999), *Rag2*^−/−^ (Jax stock 008309) (Hao and Rajewsky, 2001), CD45.1 (Jax stock 002014) (Janowska-Wieczorek et al., 2001; Schluns et al., 2002; Shen et al., 1985; Yang et al., 2002), *Cγ1*^Cre^ (Jax stock 010611) (Casola et al., 2006), and *Rosa26-YFP*^LSL^ (Jax stock 006148) (Srinivas et al., 2001), J_H_T (Jax stock 002438) (Gu et al., 1993), and OT-II (Jax stock 004194) (Barnden et al., 1998) mice were obtained from the Jackson Laboratory. *Eif3e*^fl/fl^, *Cγ1*^Cre^, and *Rosa26-YFP*^LSL^ mice were crossed to generate *Eif3e*^fl/fl^*Cγ1*^Cre^*Rosa26-YFP*^LSL^ (cKO) mice. *MHC II*^−/−^ (M2KO) and *Eif3e*^fl/fl^*Cγ1*^Cre^*Rosa26-YFP*^LSL^ mice were crossed to generate *MHC II*^−/−^*Eif3e*^fl/fl^*Cγ1*^Cre^*Rosa26-YFP*^LSL^ (M2KO;cKO) mice. Cohorts of mice were monitored for disease development for up to 1.5 years and euthanized for analysis when they appeared sick. Littermate control mice were euthanized at the same time as experimental control and were counted as “right-censored” data in the Kaplan–Meier survival analysis (Stel et al., 2011). Macroscopically, examination was performed for mice of various genotypes at the age of 5–26 wk before they appeared sick. All mice were bred and housed in specific pathogen-free facilities under a 12-h light–dark cycle. All animal experiments were approved by the Animal Care and Use Committee of Xiamen University.

### Flow cytometry

Single-cell suspensions were prepared from freshly harvested tissues after lysis of erythrocytes. 1–4 × 10^6^ cells were stained with antibody cocktails in PBS containing 0.5% BSA. For intracellular cytokine staining, cells were stimulated with PMA and ionomycin in the presence of BD GolgiPlug (555029; BD Biosciences) for 4 h at 37°C. Following surface staining, intracellular cytokine staining was performed with Cytofix/Cytoperm Fixation/Permeabilization Kit (554714; BD Biosciences) following the manufacturer’s instructions. Transcriptional factor staining for Foxp3 and Bcl6 was performed using the Fixation/Permeabilization kit (00-5123-43 and 00-8333-56; eBioscience) following the manufacturer’s instructions. For staining of CXCR5 (Liu et al., 2021), single cells were stained with purified antibody against mouse CXCR5 (2G8; BD), followed by biotin-conjugated antibody to rat IgG2a (MRG2a-83; BioLegend) and streptavidin-APC. All flow cytometry data were acquired on LSRFortessa X-20 (BD Biosciences) or a NovoCyte flow cytometer (ACEA Biosciences), and analyzed with FlowJo software 10.

### Lymphoma transplantation

Total cells (5 × 10^6^) from involved organs, including the spleens, lymph nodes, white nodes in the livers and the lungs, of sick cKO mice were resuspended in 500 μl PBS and adoptively transferred into 2- to 4-mo-old *Rag2*^−/−^ mice. Recipient mice were monitored for up to 3 mo for lymphoma development and euthanized for analysis when they appeared sick.

### Single-cell RNA-seq

Each sample for single-cell sequencing was prepared by pooling cells from 2 to 3 control or cKO mice, with two independent biological replicates for each group. The spleens and lymph nodes of 2-mo-old control and cKO mice were harvested, washed in ice-cold PBS (SH30256.01; Hyclone), and dissociated using SeekMate Tissue Dissociation Reagent Kit A Pro (K01801301; SeekGene) or SeekMate Tissue Dissociation Kit C (K01501; SeekGene) from SeekGene following the manufacturer’s instructions. Samples were treated with DNase I (9003-98-9; Sigma-Aldrich) and analyzed by Fluorescence Cell Analyzer (Countstar Rigel, S2) or SeekMate Tinitan Fluorescence Cell Counter (M002C; SeekGene) with AO/PI reagent after lysis of erythrocytes (R1010; Solarbio) to measure cell counts and viability. Dead cells and debris were removed using Dead Cell Removal Kit (130-109-398/130-090-101; Miltenyi). Cells were then washed twice with RPMI 1640 (11875119; Gibco) and resuspended in RPMI 1640 with 2% FBS (10100147C; Gibco) at 1 × 10^6^ cells per ml for further sorting or single-cell sequencing library construction. Cells were sorted for total live cells or YFP^+^ cells on BD Aria III or BD Aria Fusion (BD Biosciences). White nodules from sick cKO or *Rag2*^−/−^ mice transplanted with tumor cells were preserved in tissue storage solution and shipped to SeekGene for preparing single-cell suspension and dead cell removal. The samples were then processed as described above and used directly for scRNA-seq and V(D)J-seq library construction.

scRNA-seq libraries were prepared using the SeekOne DD Single Cell 3′ library preparation kit (Cat No. K00202; SeekGene) following the manufacturer’s instructions. The indexed sequencing libraries were cleaned up with VAHTS DNA Clean Beads (N411-01; Vazyme) and analyzed by Qubit (Q33226; Thermo Fisher Scientific) and Bio-Fragment Analyzer (Qsep400; BiOptic). The libraries were then sequenced on Illumina NovaSeq 6000 with PE150 read length or DNBSEQ-T7 platform with PE150 read length.

### Single-cell V(D)J-seq library construction, sequencing, and analysis

Single-cell V(D)J-seq libraries were constructed using the SeekOne DD Single Cell 5′ library preparation kit and SeekOne DD Single Cell V(D)J Enrichment Kit (mouse TCR&BCR, Cat No. K01101&K01201; SeekGene) following the manufacturer’s instructions. The indexed sequencing libraries were cleaned up with VAHTS DNA Clean Beads (N411-01; Vazyme) and analyzed by Qubit (Q33226; Thermo Fisher Scientific) and Bio-Fragment Analyzer (Qsep400; BiOptic). The libraries were then sequenced on Illumina NovaSeq 6000 with PE150 read length or DNBSEQ-T7 platform with PE150 read length. For TCR-sequencing data analysis, MiTCR and VDJtools were used for analysis of hundreds of millions of raw high-throughput sequencing reads containing sequences encoding mouse α or β TCR chains (Bolotin et al., 2013; Shugay et al., 2015). BCR-sequencing data were analyzed following the instructions of MiXCR (Bolotin et al., 2015).

### scRNA-seq data analysis

The data from two independent biological replicates were merged for analysis. scRNA-seq data were processed and analyzed using the Seurat package (v4.3.0) (Butler et al., 2018). Quality control was performed to remove low-quality cells, excluding those with <201 UMIs, <101 expressed genes, or over 10% of UMIs derived from mitochondrial genes. Raw count matrices were first normalized using the “LogNormalize” method to scale and normalize gene expression values. Highly variable genes (*n* = 2,000) were identified using the FindVariableFeatures function, followed by data scaling with the ScaleData function. Principal component analysis (PCA) was performed with RunPCA to reduce the dimensionality of the data. To address batch effects across datasets derived from different samples, Harmony (v0.1.0) (Korsunsky et al., 2019) was applied for integration and batch correction based on the PCA-reduced dimensions. The Harmony-corrected embeddings were then used for downstream clustering and Uniform Manifold Approximation and Projection visualization. Specifically, the FindNeighbors function was used to construct a shared nearest neighbor graph, and clusters were identified using the FindClusters function. For cell-type annotation, marker genes for each cluster were identified using the R package COSG (v0.9.0) (Dai et al., 2022), which computes cluster-specific marker genes with high resolution. Identified marker genes were subsequently used to assign cell-type identities to clusters based on prior biological knowledge and established marker gene databases.

### Single-cell BCR-sequencing and TCR-sequencing analysis

Raw sequencing data were processed using the SeekSoulTools vdj (v1.2.1) to assemble full-length V(D)J sequences and identify CDR3 regions. Using barcode and raw clonotype ID information, productive clonotypes were filtered. Processed FASTA sequences were further annotated using the IMGT database.

### GSEA of DEGs

We firstly extracted the differential expression gene sets between wild-type and cKO cell types. GSEA was employed to analyze the enriched terms of those gene sets by using KEGG gene sets (Mootha et al., 2003; Subramanian et al., 2005).

### Bone marrow reconstitution

To generate chimeras without CD4^+^ T cells, bone marrow cells from control or cKO mice were adoptively transferred into lethally irradiated (6.5 Gray) CD45.1 or MHC II^−/−^ mice. To generate chimeras with B cell–specific deletion of MHC II, bone marrow cells from control, cKO, M2KO, or M2KO;cKO mice were mixed with bone marrow cells from J_H_T mice at a 1:4 ratio and adoptively transferred into lethally irradiated (4.5 Gray) Rag2^−/−^ mice. In all experiments, a total number of 5 million bone marrow cells were transferred into each recipient. Recipient mice were analyzed at 3–4 mo after reconstitution.

### Histology

For hematoxylin and eosin staining, tissues were fixed subsequently in 4% formaldehyde (HT501128; Sigma-Aldrich) and 70% ethanol, embedded in paraffin, sectioned, and stained with hematoxylin and eosin. For immunofluorescence, spleens and draining lymph nodes from control and cKO mice were fixed overnight at 4°C in a fixative solution consisting of BD Fix/Perm Solution (Cat No. 51-2090KZ) and PBS at a 1:2 ratio. The fixative solution was discarded, and the fixed tissues were washed with PBS for 10 min on a shaker. 30% sucrose (Sigma-Aldrich) was added to tissues, which were then incubated at 4°C overnight. The tissues were subsequently embedded in OCT (Tissue-Tek; Sakura). Tissue sections were cut at a thickness of 10 μm with a Leica CM1950 machine. The sections were immunostained for CD4, B220, and GL7 at 4°C in a dark box for 12 h. Images were acquired by using a Leica TCS SP8 confocal microscope (Leica) and analyzed by Imaris software.

### Primary lymphocyte purification

B and CD4^+^ T cells were purified from white nodules of sick cKO mice or *Rag2*^−/−^ mice transplanted with primary tumor cells by positive selection with BD IMag Beads (557812; BD) using biotinylated antibodies (CD19 or CD4) (BioLegend). Naïve B cells from the spleen of control or cKO mice were purified by negative selection with Beaver Beads (22307; Beaver) and biotinylated antibodies (CD43, CD9, CD5, TER119, and CD93) (eBioscience and BioLegend). Naïve CD4^+^ T cells from the spleen and lymph nodes of OT-II mice were purified by negative selection with Beaver Beads and biotinylated antibodies (CD19, CD44, CD25, CD8, TCRγ/δ, NK1.1, CD11c, CD11b, TER119, F4/80, Gr1, and CD105) (BioLegend).

### iGCB culture

Naïve B cells were cultured in 6- or 12-well plates (Thermo Fisher Scientific) for 4 days with 40LB cells irradiated with 120 Gy in the presence of IL-4, in 1640 complete medium supplemented with 10% FBS, 5.5 × 10^−5^ M β-mercaptoethanol, 10 mM HEPES, 1 mM sodium pyruvate, 100 U/ml penicillin, and 100 μg/ml streptomycin (Gibco), 1× NEAA (Gibco), and 1 ng/ml IL-4 (Noveprotein, CK74) (Nojima et al., 2011). YFP^+^ iGCBs were sorted on BD Aria Fusion (BD Biosciences) for RNA-seq analysis. Harvested iGCBs were stained for 7 min at room temperature for FACS analysis. For iGCB proliferation analysis, naïve B cells were labeled with CellTrace Violet (C34557; Thermo Fisher Scientific) at 37°C for 20 min following the manufacturer’s instruction, cultured in the iGCB system for 3 days, and analyzed by flow cytometry for CellTrace Violet intensity. For cell death analysis, harvested iGCBs were stained with Annexin V and 7-AAD (559763; BD) following the manufacturer’s instruction.

### B–T coculture

Day 4 iGCBs were purified by removing 40LB cells through negative selection with an anti-H-2Kd antibody (Haniuda and Kitamura, 2019), resuspended at 3.3 × 10^6^ cells/ml in prewarmed RPMI 1640 complete medium, loaded with 0 or 4 nM OVA peptide 323–339, and supplemented with either anti-MHC II (isotype control IgG2b, 8 μg/ml), anti-CD80 (isotype control IgG2a, 8 μg/ml), or anti-OX40L (isotype control IgG2b, 40 μg/ml) blocking antibodies (BioLegend). The cell suspension was added to round-bottom 96-well plates at 100 μl per well and incubated in a 37°C CO_2_ incubator for 1 h. Naïve CD4^+^ T cells from OT-II mice were labeled with CellTrace Violet and resuspended in prewarmed RPMI 1640 complete medium at a density of 1.1 × 10^6^ cells/ml. To each well containing OVA peptide–loaded iGCBs, 150 μl of CellTrace Violet–labeled OT-II naïve CD4^+^ T cells was added. After thorough resuspension, plates were placed in a 37°C CO_2_ incubator for 2.5 days, followed by flow cytometry analysis.

### Antibodies and reagents

For flow cytometry analysis: Anti-Bcl-6 (K112-91), anti-CD95 (Jo2), anti-CD138 (281-2), and anti-IL-4 (BVD4-1D11) antibodies were purchased from BD. Anti-B220 (RA3-6B2), anti-CD19 (1D3), anti-CD80 (16-10A1), anti-CD3e (145-2C11), anti-CD4 (GK1.5), anti-CD4 (RM4-5), anti-CD8a (53-6.7), anti-CD44 (IM7), anti-CD62L (MEL-14), anti-CD45.1 (A20), anti-CD45.2 (104), anti-MHC II (M5/114.15.2), anti-PD-1 (J43), anti-IgD (11-26c (11-26)), anti-IFN-γ (XMG1.2), anti-streptavidin, anti-CD70 (FR70), anti-CD40 (1C10), anti-OX40L (RM134L), and anti-ICAM-1 (YN1/1.7.4) antibodies were purchased from Thermo Fisher Scientific. Anti-CD86 (GL-1), anti-GL7 (GL7), anti-CD16/32 (93), and anti-CD275 (HK5.3) antibodies were purchased from BioLegend.

For cell purification: Anti-CD5 (53-7.3), anti-CD43 (1B11), anti-TER-119 (TER-119), anti-CD19 (6D5), anti-CD4 (RM4-5), anti-CD44 (IM7), anti-CD25 (PC61), anti-CD8a (53-6.7), anti-TCRγ/δ (GL3), anti-NK1.1 (PK136), anti-CD11c (N418), anti-CD11b (M1/70), anti-F4/80 (BM8), anti-Gr1 (RB6-8C5), anti-CD105 (MJ7/18), and anti-H-2Kd (SF1-1.1) antibodies were purchased from BioLegend. Anti-CD93 (AA4.1) antibody was purchased from Thermo Fisher Scientific. Anti-CD9 (KMC8) antibody was purchased from BD.

For B–T coculture assays: anti-MHC II (M5/114.15.2), anti-CD80 (W17200C), anti-OX40L (RM134L), rat IgG2a control, and rat IgG2b control antibodies were purchased from BioLegend.

For immunoblot: anti-eIF3e antibody was purchased from Abcam, and anti-β-actin (8H10D10) antibody was purchased from CST.

### RNA-seq and data analysis

Naïve B cells from control and cKO mice were cultured in the iGCB system for 3 days. YFP^+^ iGCBs were sorted by flow cytometry. Total RNA was extracted using RNeasy Kit (QIAGEN) following the manufacturer’s instructions. RNA samples were sent to Novogene for sequencing. The clean data, with adapter sequences trimmed and low-quality reads removed, were used for downstream analysis. The clean data were processed to read counts following the Hisat2-StringTie pipeline (Pertea et al., 2016). Using prepDE.py provided by StringTie, read count information was extracted for differential analysis conducted by DESeq2 (Love et al., 2014). GO clustering analysis of DEGs was performed with Metascape (Zhou et al., 2019). Volcano plots and bubble plots were generated at https://www.bioinformatics.com.cn, an online platform for data analysis and visualization (Tang et al., 2023).

### Protein sample preparation for mass spectrometry

YFP^+^ iGCBs at day 3 were sorted by flow cytometry and processed using gel-aided sample preparation for mass spectrometry. Briefly, 50 μl samples were reduced with 10 mM TCEP, alkylated with 10 mM iodoacetamide, and mixed with an equal volume of 30% acrylamide/bis-acrylamide solution. Polymerization was initiated by adding 2 μl TEMED and 2 μl 10% APS, and the gel was allowed to solidify at RT for 20 min. The solidified gel was cut into pieces and fixed in 1 ml methanol/acetic acid/water (50:40:10) for 10 min with mixing. After pulse centrifugation, the supernatant was discarded, and gel pieces were washed with 1 ml 100 mM NH_4_HCO_3_ for 10 min with rotation. Dehydration was achieved by adding 1 ml acetonitrile (ACN), and this NH_4_HCO_3_/ACN wash cycle was repeated three times. Gel pieces were then dried with 500 μl ACN until agglomerated. Trypsin solution (100 μl, 20 μg/μl) was added, and digestion proceeded overnight at 37°C. Peptides were extracted by adding 100 μl extraction buffer (5% formic acid/ACN, 1:2 vol/vol) and incubating at 37°C for 15 min with shaking, followed by adding 200 μl ACN. The supernatant was collected, and gel pieces were rehydrated in 5% formic acid, then dehydrated again with 200 μl ACN. Supernatants were pooled after an additional round of dehydration with 50 μl ACN. Samples were dried in a vacuum concentrator and resuspended in 0.1% FA for LC-MS analysis.

### LC-MS/MS

Peptides were analyzed on an EASY-nLC 1000 UHPLC system coupled to an Orbitrap Fusion mass spectrometer (Thermo Fisher Scientific). Mobile phase A was 0.1% FA/2% ACN in H_2_O, and mobile phase B was 0.1% FA/2% H_2_O in ACN. Samples were loaded onto a 100 μm I.D. × 150 mm analytical column (in-house–packed with 1.9 μm C18 resin, Dr. Maisch) and separated using a 120-min gradient at 300 nl/min (B: 3–8% in 8 min, 8–20% in 85 min, 20–40% in 17 min, 40–95% in 5 min, 95% for 5 min). Data were acquired in a data-dependent mode. MS1 scans were performed in the Orbitrap at 120,000 resolution over 300–1,800 m/z, with an AGC target of 5 × 10^5^. Precursor ions with charge 2–8 were selected for MS/MS. MS2 scans were acquired in the ion trap over 100–1,800 m/z, with an AGC target of 1 × 10^4^ and a maximum injection time of 200 ms.

### Integrative analysis of transcriptome and proteome data

To investigate posttranscriptional and translational regulatory effects, we performed an integrative analysis of the matched RNA-seq and unlabeled quantitative mass spectrometry datasets. The analysis was restricted to genes exhibiting statistically significant changes in both datasets (RNA-adjusted P <0.05 and MS Q <0.05) and an absolute log2FC >0.3 in at least one dataset. Because steady-state protein levels are predominantly driven by transcript abundance, we uncoupled translational regulation from transcriptional changes using a linear regression model (Protein log_2_FC ∼ RNA log_2_FC). Genes with a standardized residual (Z-score) >1.2 were defined as significant translational outliers. To characterize the specific modes of regulation, these outliers were classified into distinct functional categories: Opposite (RNA and protein abundance changed in biologically opposing directions), Intensified (protein FC exceeded RNA FC by >0.5 log_2_ units), and Buffered (protein response was blunted by >0.5 log_2_ units compared with the RNA shift). To identify pathway-specific regulatory patterns, lists of significantly differentially expressed proteins were submitted to the Metascape web portal for functional enrichment. All data manipulation and visualization were performed in R using the dplyr and ggplot2 packages.

### Immunoblot

B cells, CD4^+^ T cells, and iGCBs were lysed in lysis buffer. The cell lysates were resolved on SDS‒PAGE gels, and the proteins were transferred to PVDF membranes (Merck Millipore). The membranes were incubated overnight at 4°C with primary antibodies diluted in NCM Universal Antibody Diluent (WB500D; NCM Biotech). After washing for 45 min in TBS buffer with 0.1% Tween-20, each membrane was incubated with a horseradish peroxidase (HRP)–conjugated goat anti-rabbit or goat anti-mouse antibody (ABclonal) for 1 h at room temperature. After washing for 12 min in TBS buffer with 0.1% Tween-20, the protein bands were visualized with Immobilon Western Chemiluminescent HRP Substrate (Merck Millipore) following the manufacturer’s instructions. Images were acquired with an Amersham Imager 600 (GE Healthcare).

### Statistical analysis

Data were analyzed using unpaired two-tailed Student’s *t* test and one-way analysis of variance (ns, not significant, P > 0.05; *P < 0.05; **P < 0.01; ***P < 0.001; ****P < 0.0001) and Fisher’s exact test. Results are shown as the mean with error bars indicating ±standard deviation of the mean. Kaplan–Meier survival curves were compared using the log-rank test.

### Online supplemental material

Fig. S1 shows the percentage, number, and activation status of T and B cells in cKO mice at different ages, along with validation of eIF3e knockout efficiency *in vivo*. Fig. S2 shows scRNA-seq and functional analyses, which reveal that the cKO phenotype relies on CD4^+^ T cell interactions via MHC II. Fig. S3 shows eIF3e protein levels, phenotypic features of cKO iGCBs, and metascape analysis of DEGs linked to translation efficiency. Table S1 shows characterization of lymphoid neoplasia in cKO mice. Table S2 shows BCR and TCR clonotype analysis of dominant clones in tumors of cKO mice. Table S3 shows summary of pathological features in cKO mice. Table S4 shows comparison of immune cell expansion in *Rag2*^−/−^ mice and primary tumors. Table S5 shows DEGs from scRNA-seq analysis of T_FH_ cells from cKO mice compared with control mice. Table S6 shows DEGs at the protein level in cKO versus control day 3 iGCBs. Table S7 shows a list of 343 translationally regulated genes in cKO day 3 iGCBs. Table S8 shows RNA-seq analysis of cKO versus control day 3 iGCBs.

## Supplementary Material

Table S1 shows characterization of lymphoid neoplasia in cKO mice.

Table S2 shows BCR and TCR clonotype analysis of dominant clones in tumors of cKO mice.

Table S3 shows summary of pathological features in cKO mice.

Table S4 shows comparison of immune cell expansion in *Rag2*^−/−^ mice and primary tumors.

Table S5 shows DEGs from scRNA-seq analysis of T_FH_ cells from cKO mice compared with control mice.

Table S6 shows DEGs at the protein level in cKO versus control day 3 iGCBs.

Table S7 shows a list of 343 translationally regulated genes in cKO day 3 iGCBs.

Table S8 shows RNA-seq analysis of cKO versus control day 3 iGCBs.

SourceData F1 is the source file for Fig. 1.

SourceData F4 is the source file for Fig. 4.

SourceData FS1 is the source file for Fig. S1.

SourceData FS3 is the source file for Fig. S3.

## Data Availability

Primary sequencing data for RNA-seq, scRNA-seq, and single-cell V(D)J-seq results are publicly available via the National Center for Biotechnology Information Gene Expression Omnibus under accession numbers GSE320249, GSE320248, and GSE320525, respectively. The mass spectrometry proteomics data have been deposited to the ProteomeXchange Consortium (https://proteomecentral.proteomexchange.org) via the iProX partner repository (Chen et al., 2022; Ma et al., 2019) with the dataset identifier PXD074851. All data are available in the article itself and its supplementary materials and are also available upon request from the corresponding authors.
