# Supplementary figures and images for: eIF3e-mediated translational checkpoint maintains immune tolerance and prevents lymphoid malignancy
Source: J Exp Med. 2026 Jun 3;223(8):e20251968. doi: 10.1084/jem.20251968 (PMC13231948; doi:10.1084/jem.20251968)

Panel I

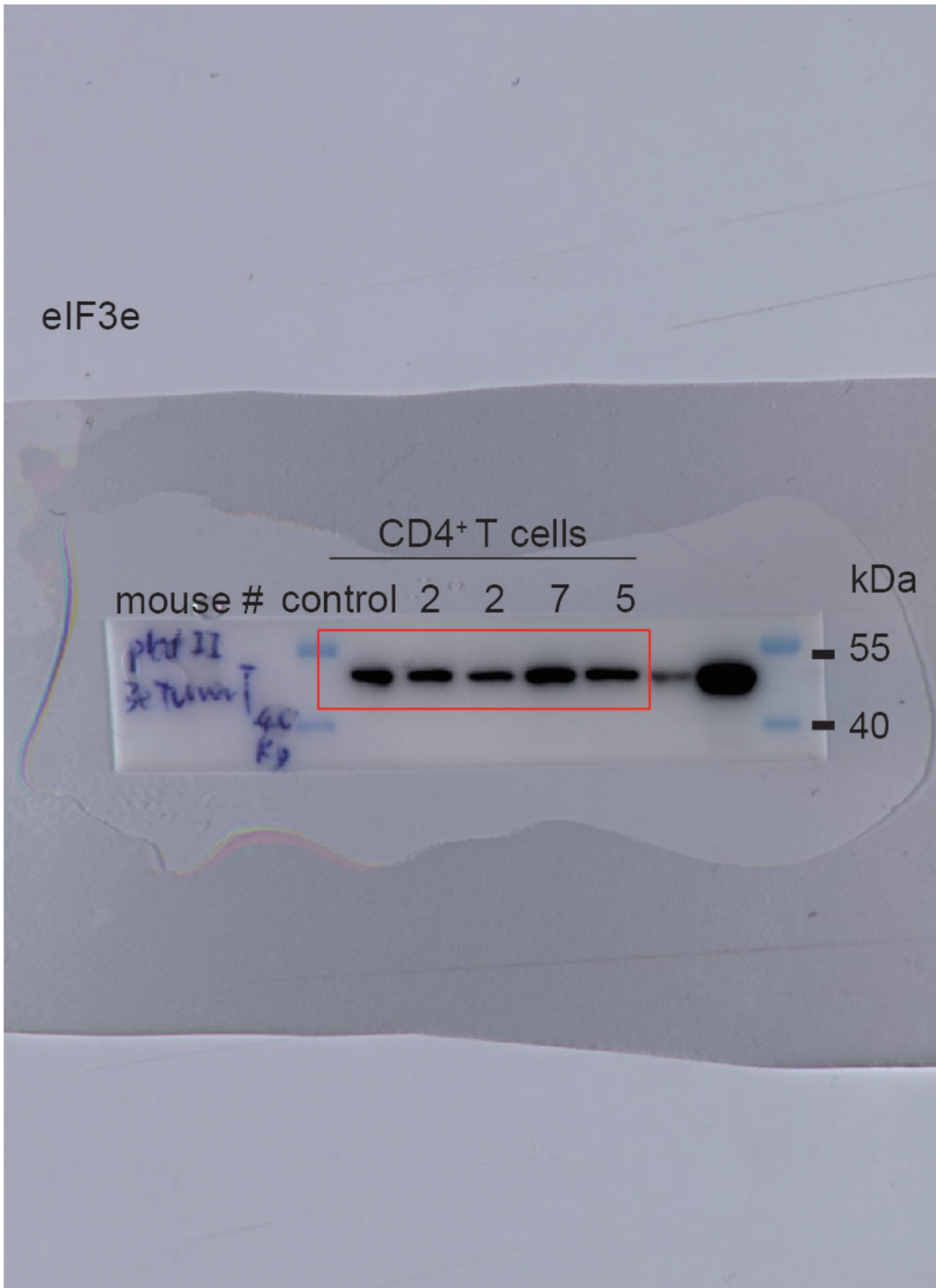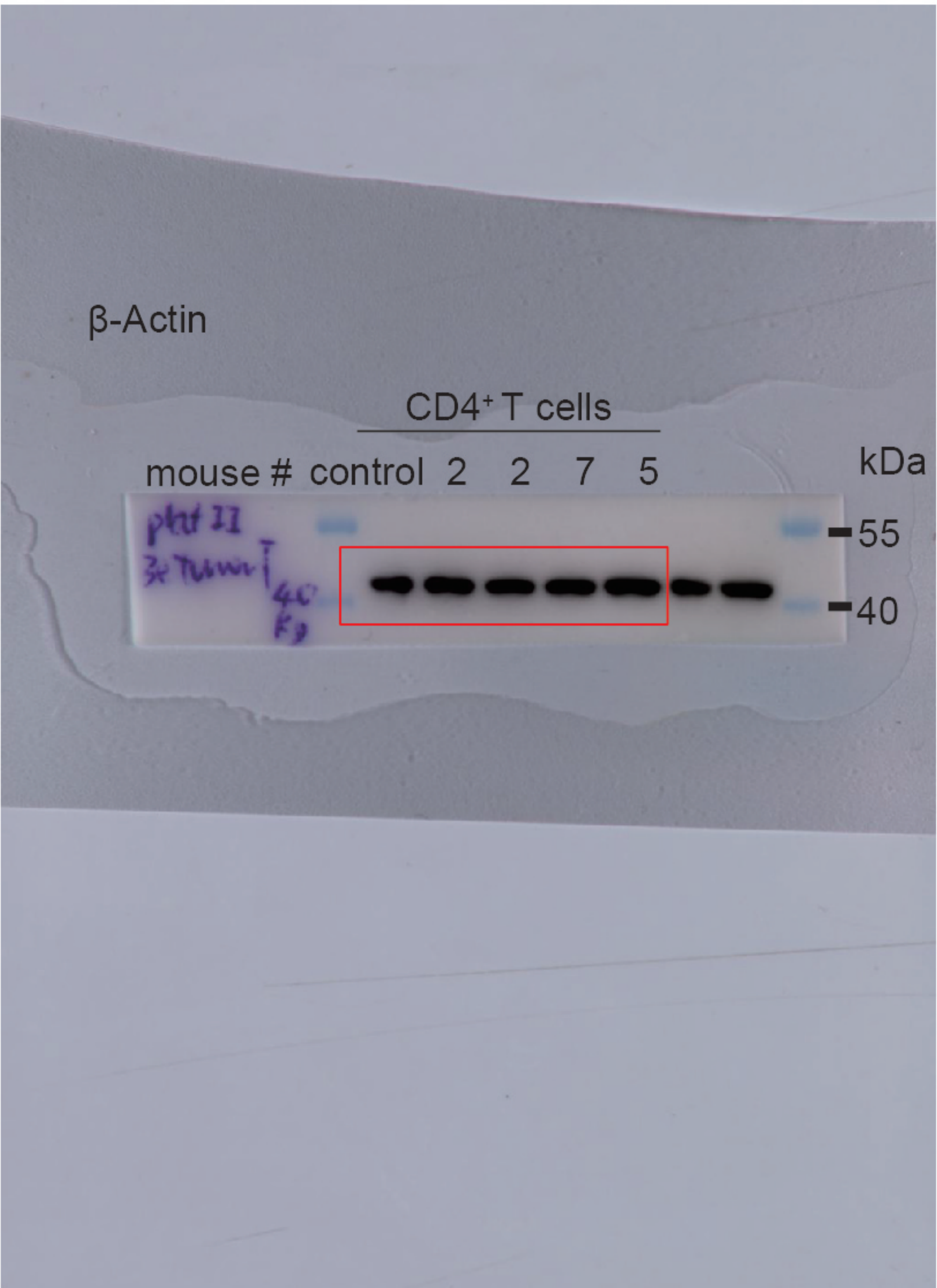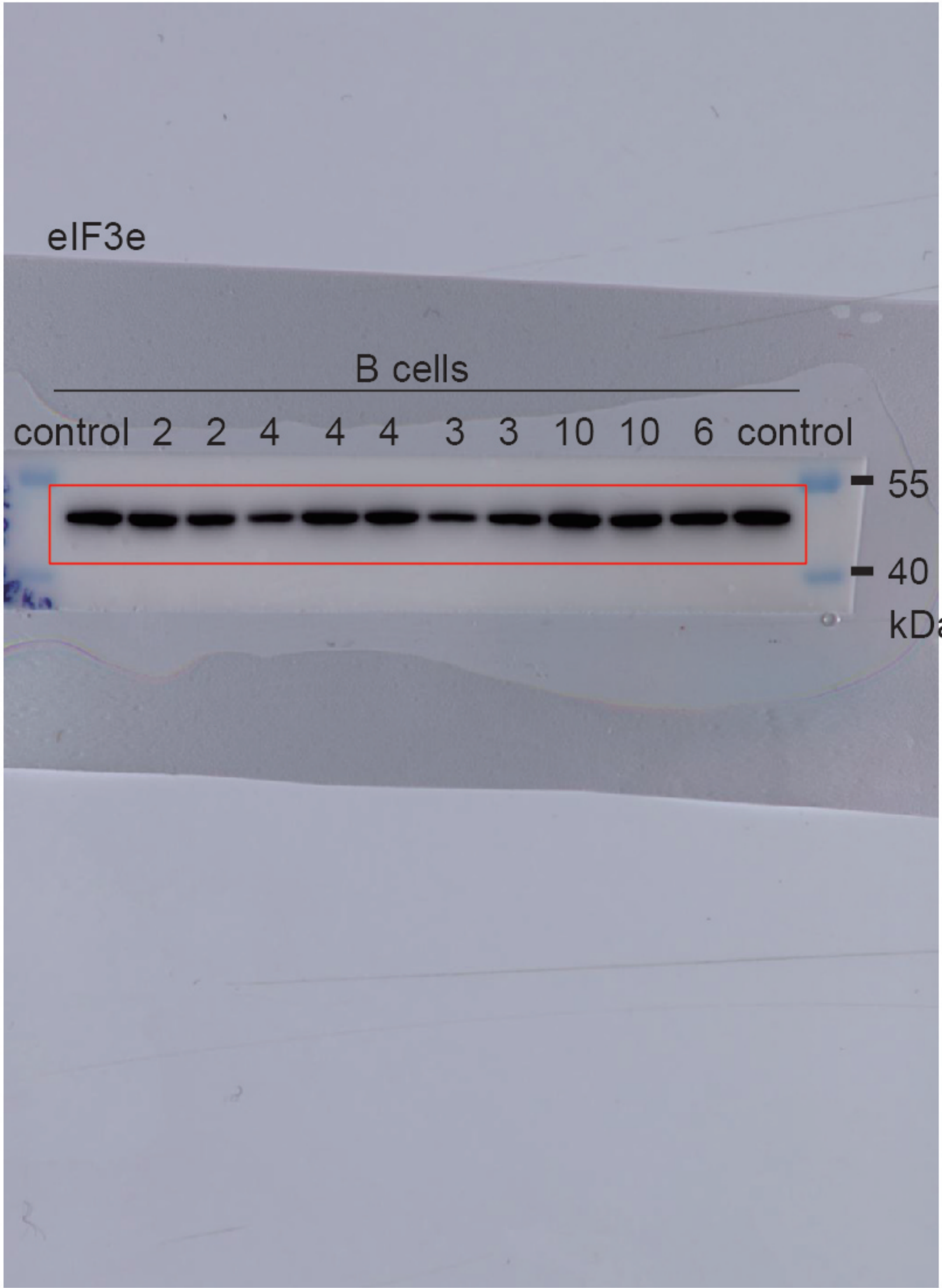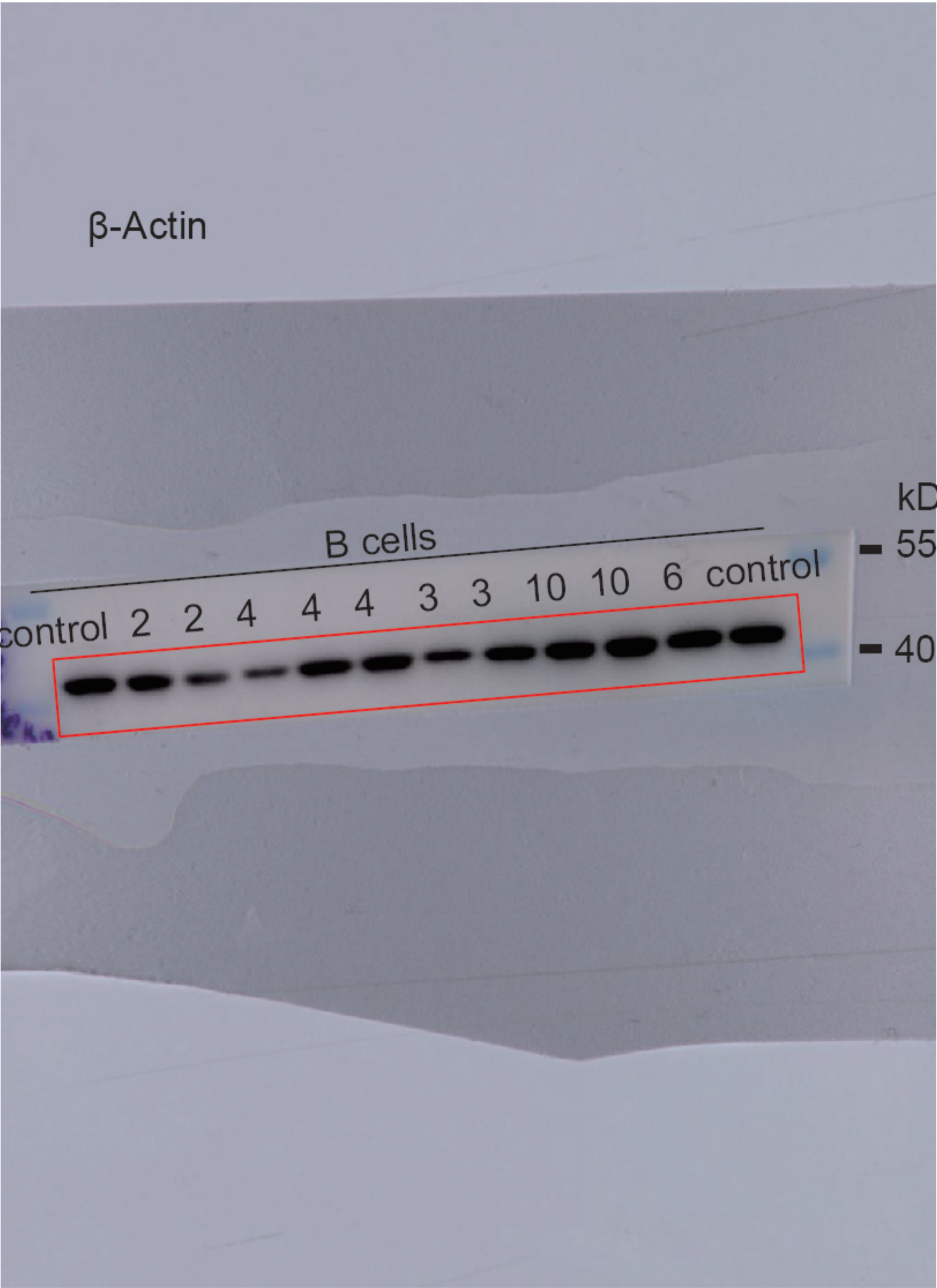

Supplement: SourceData F1 — is the source file for Fig. 1. [file jem_20251968_sourcedataf1.pdf]

Panel B

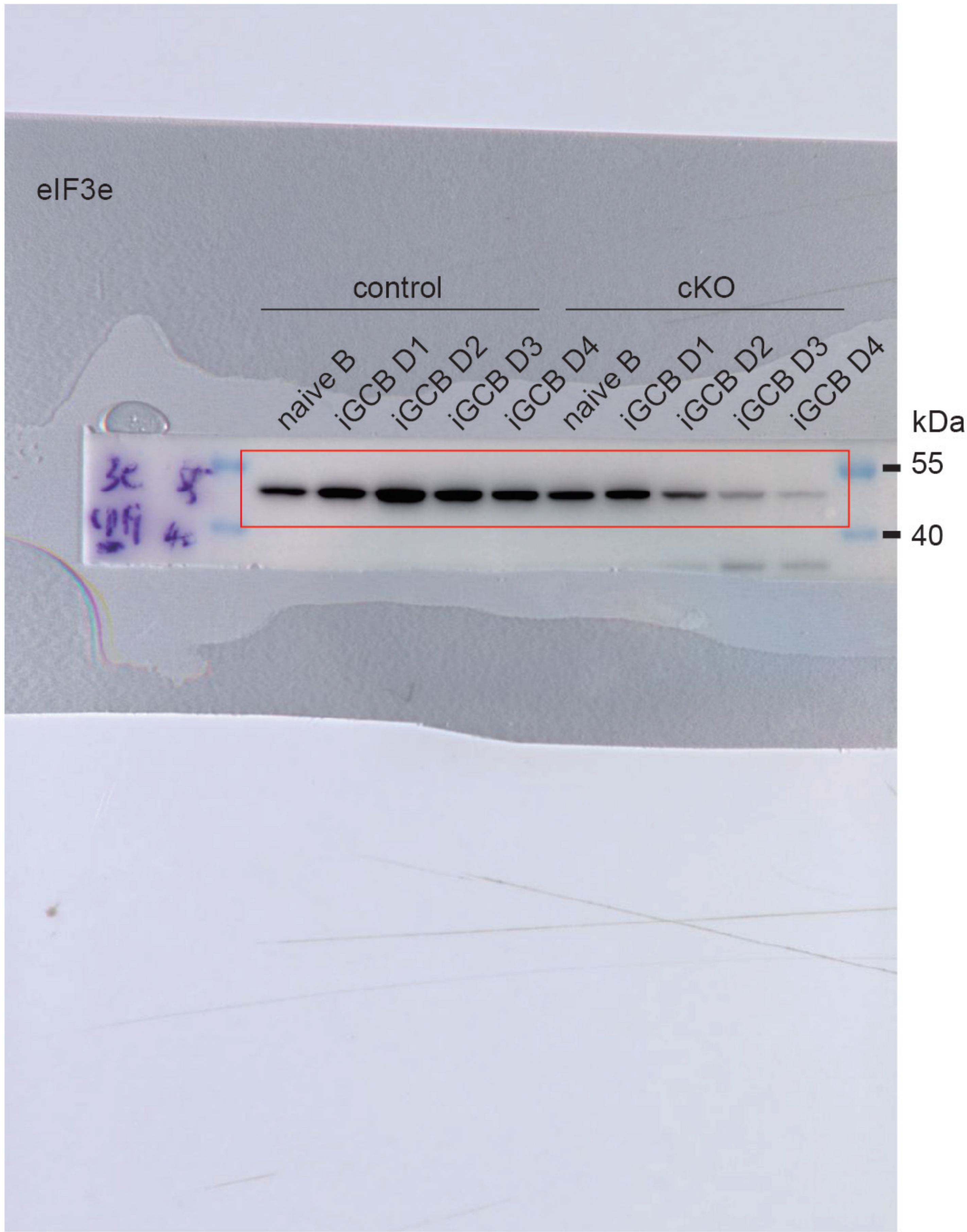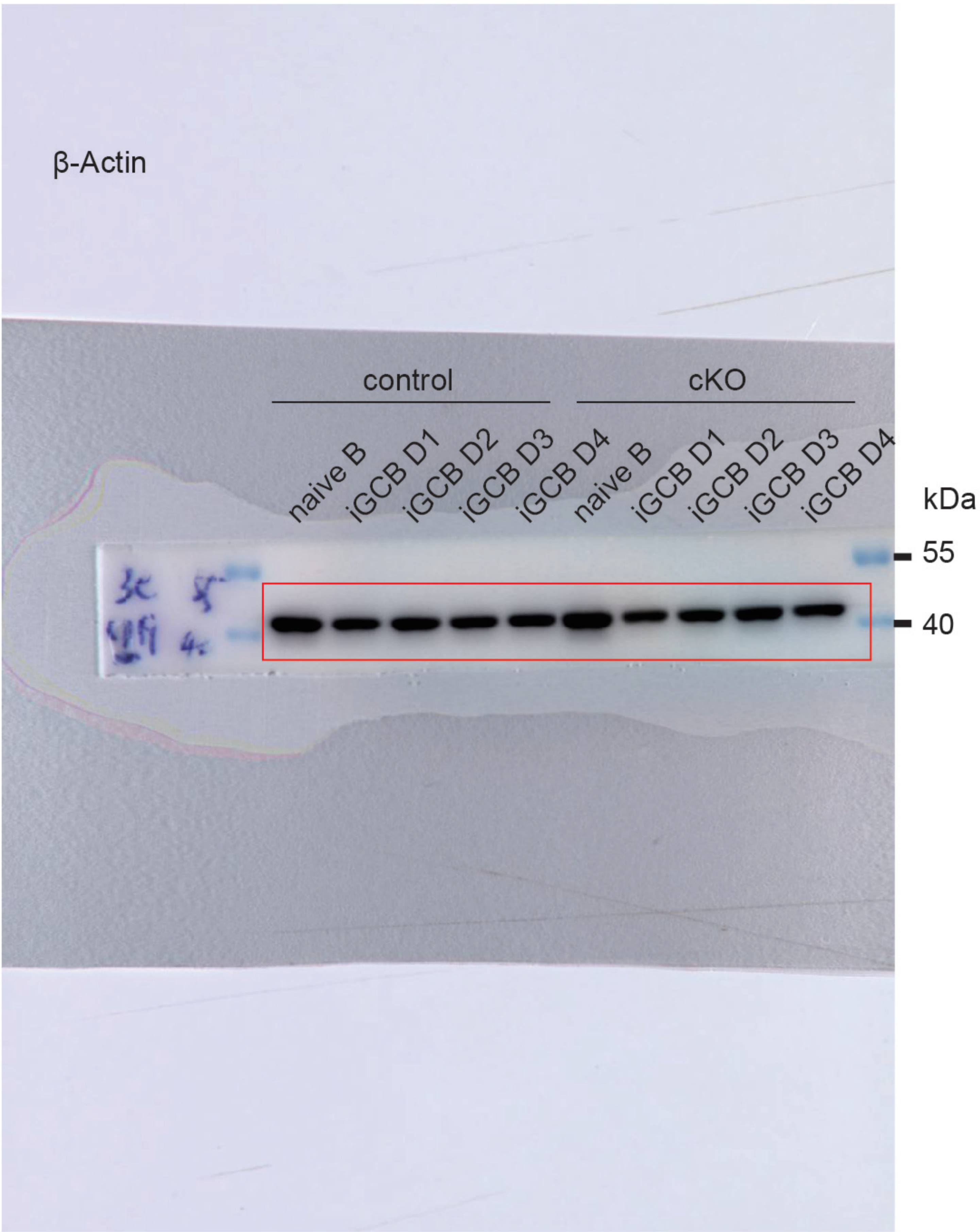

Supplement: SourceData F4 — is the source file for Fig. 4. [file jem_20251968_sourcedataf4.pdf]

Panel F

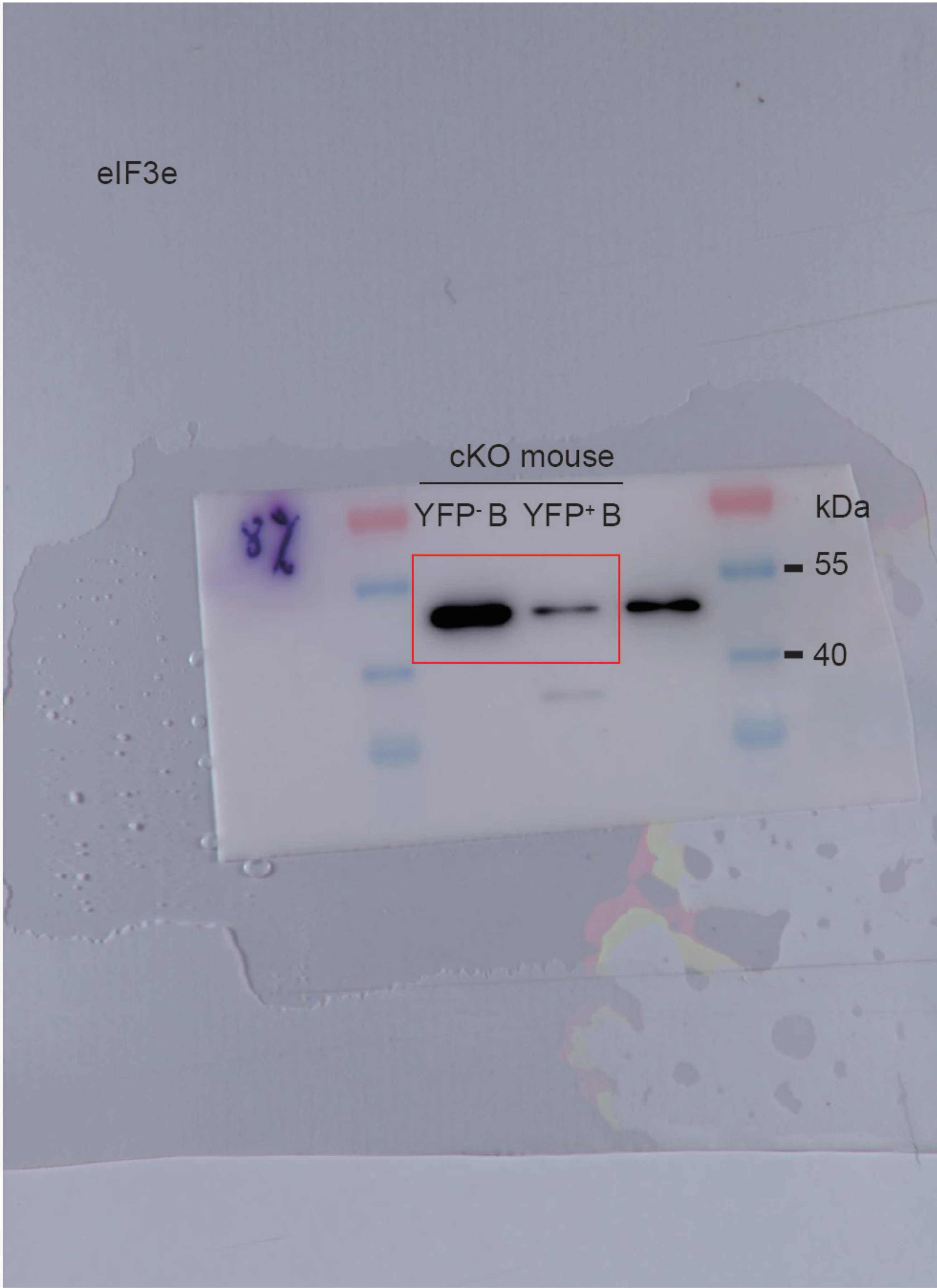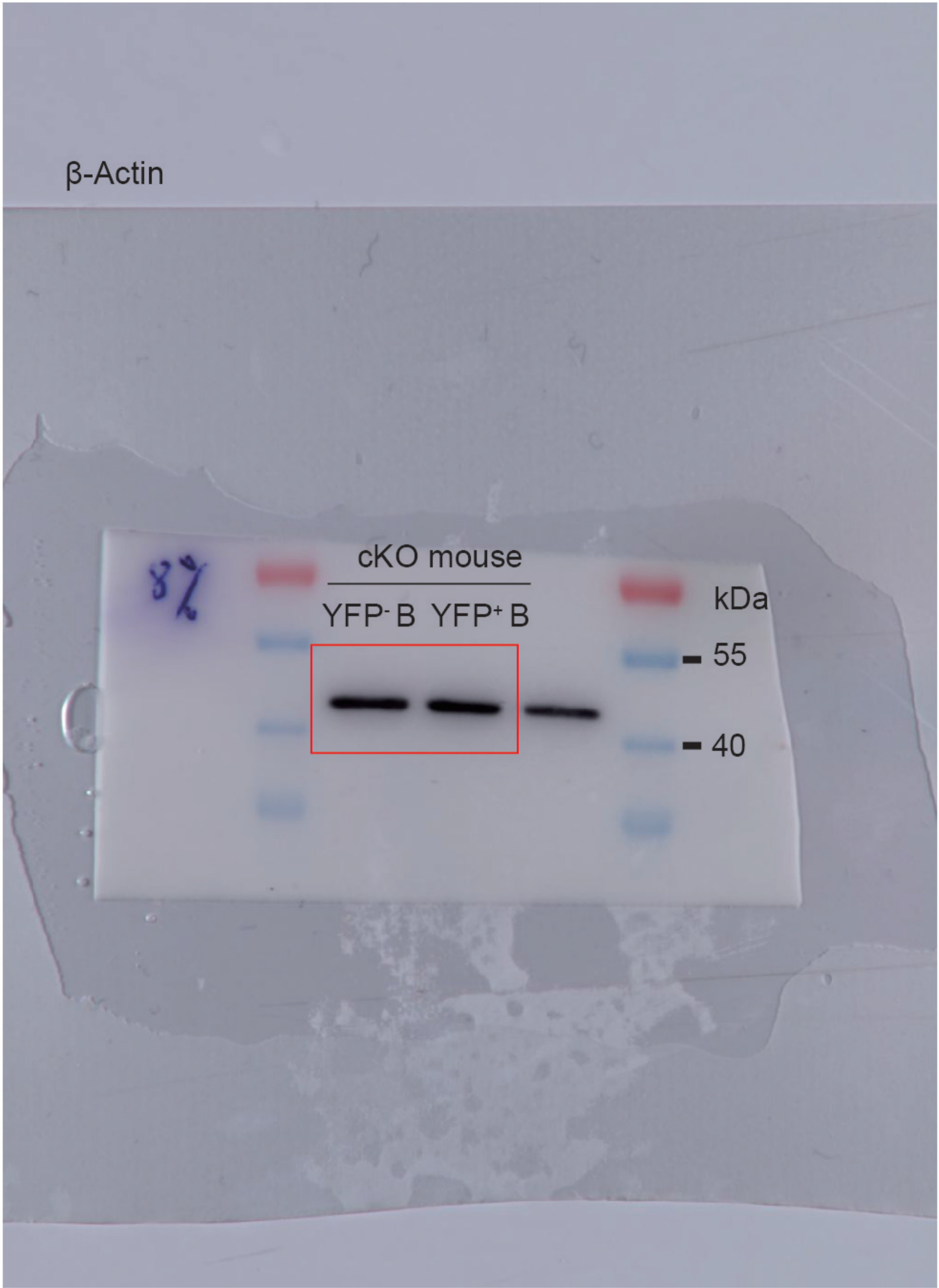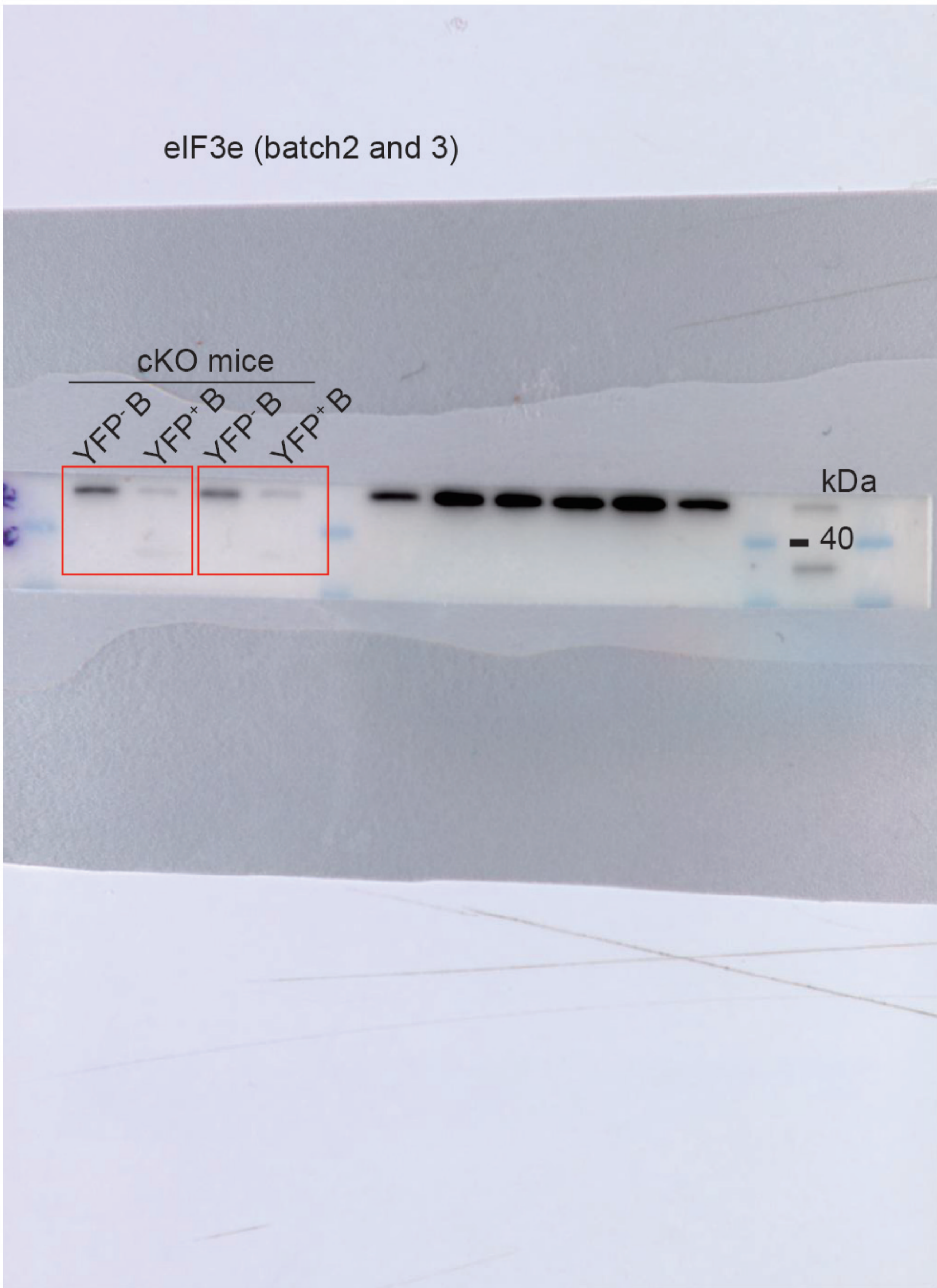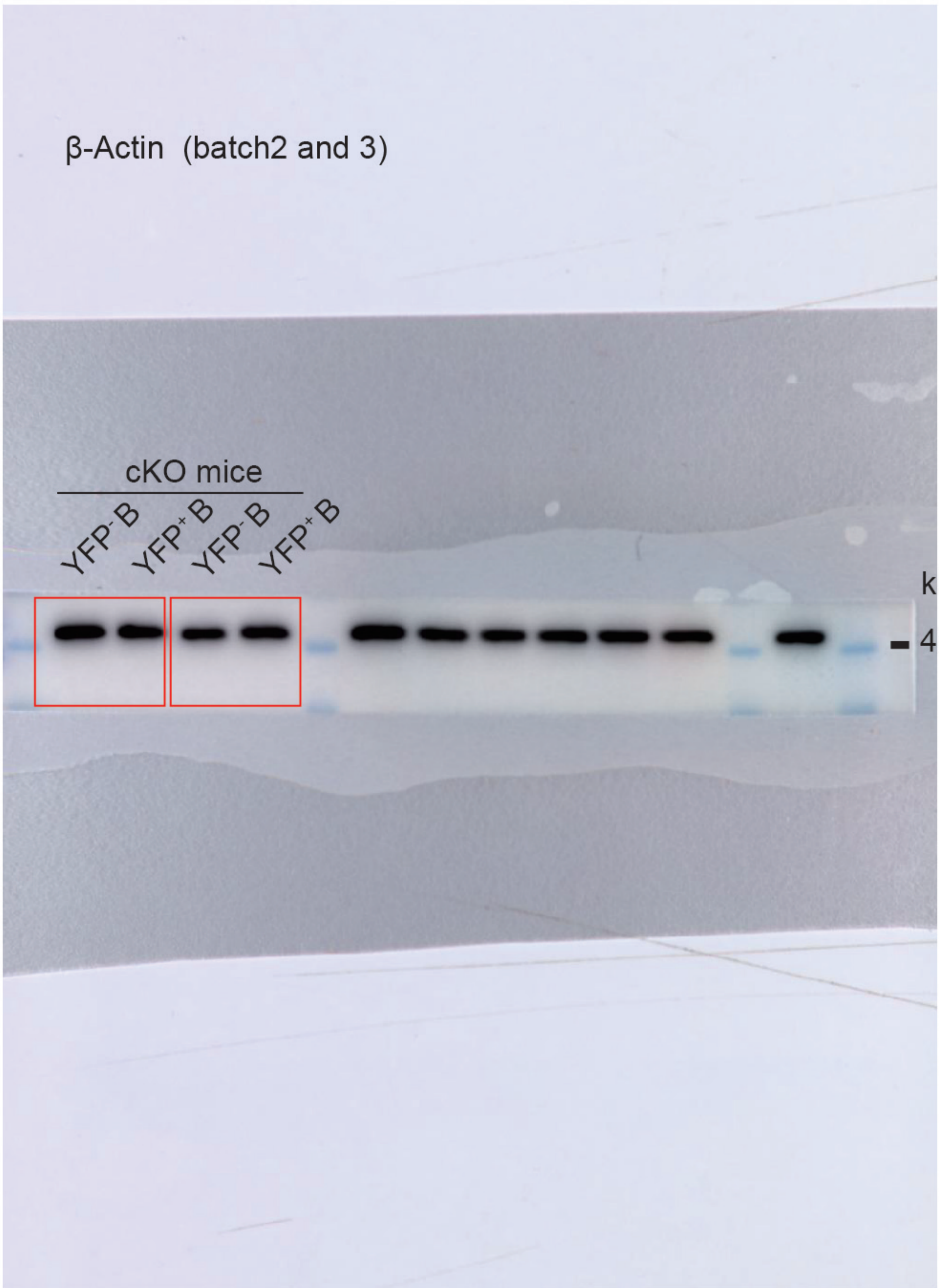

Supplement: SourceData FS1 — is the source file for Fig. S1. [file jem_20251968_sourcedatafs1.pdf]

Panel A

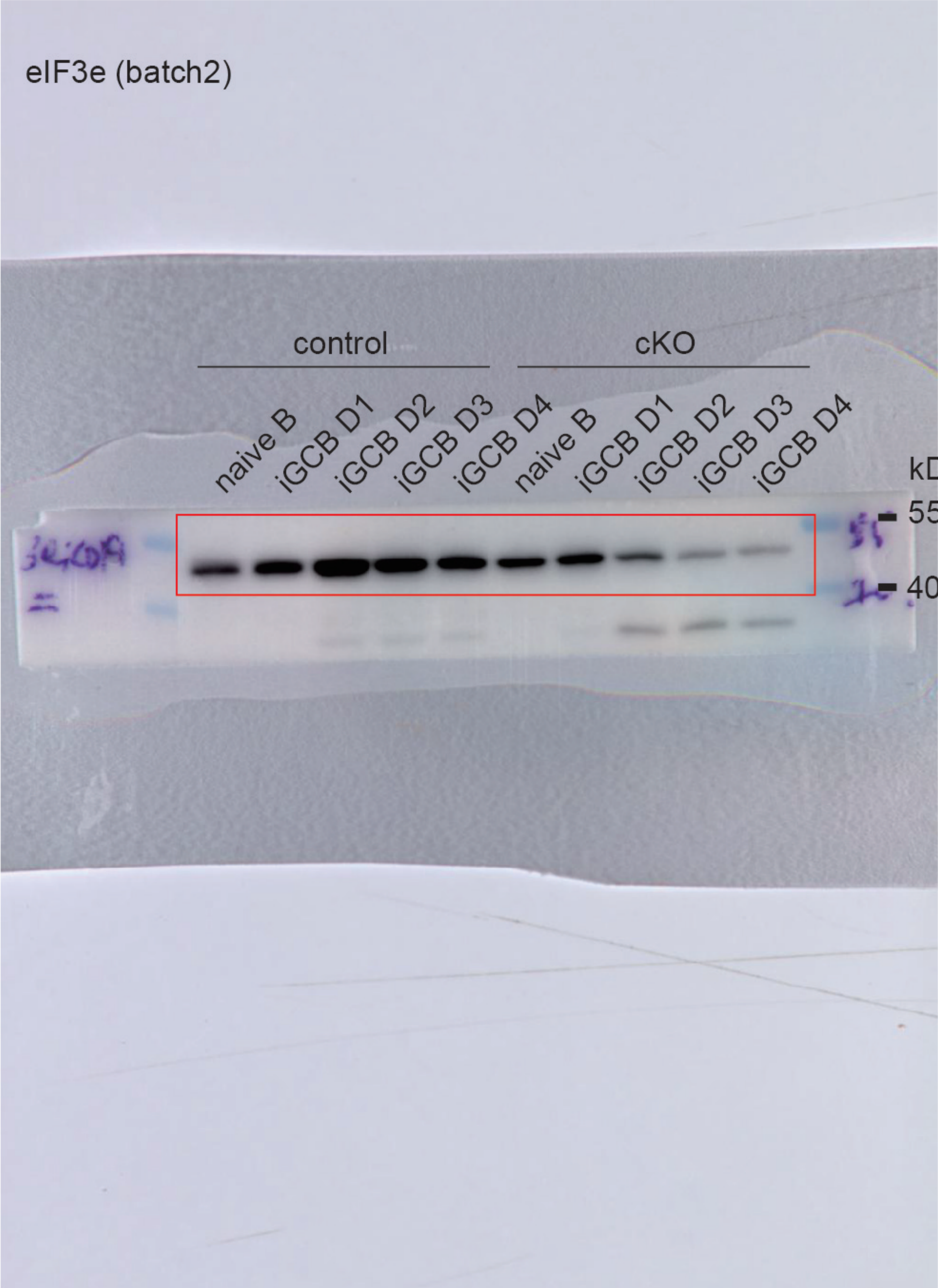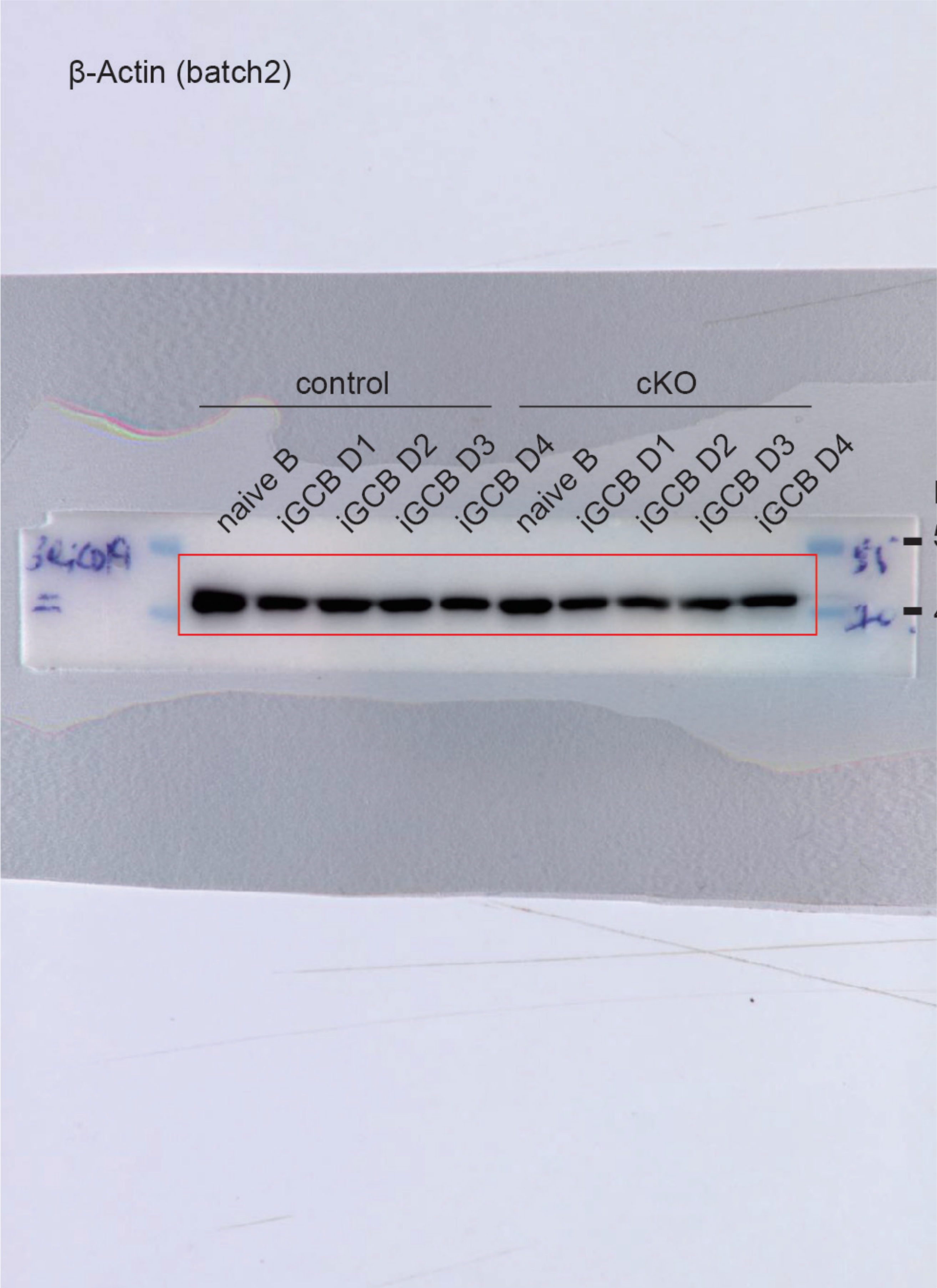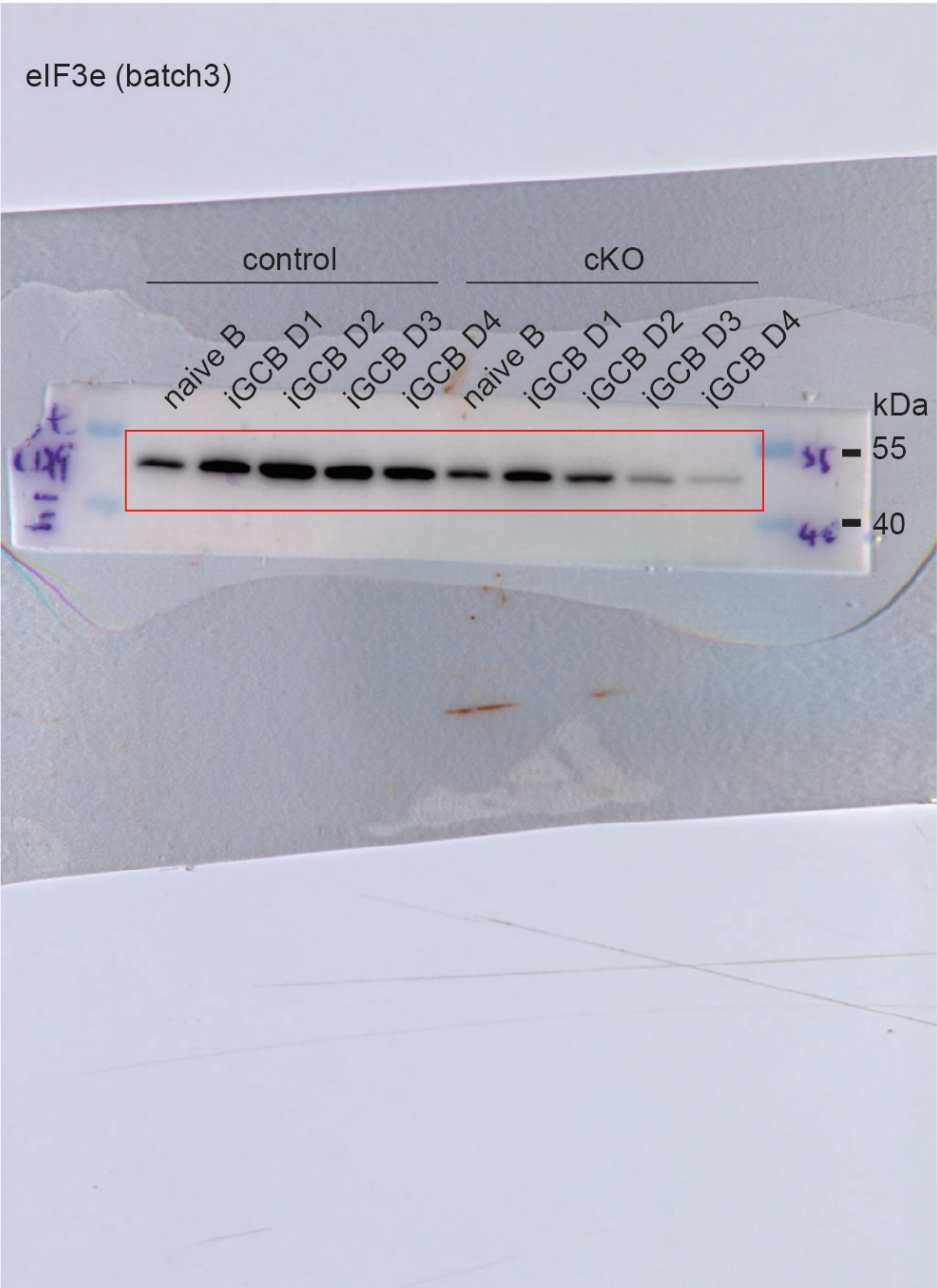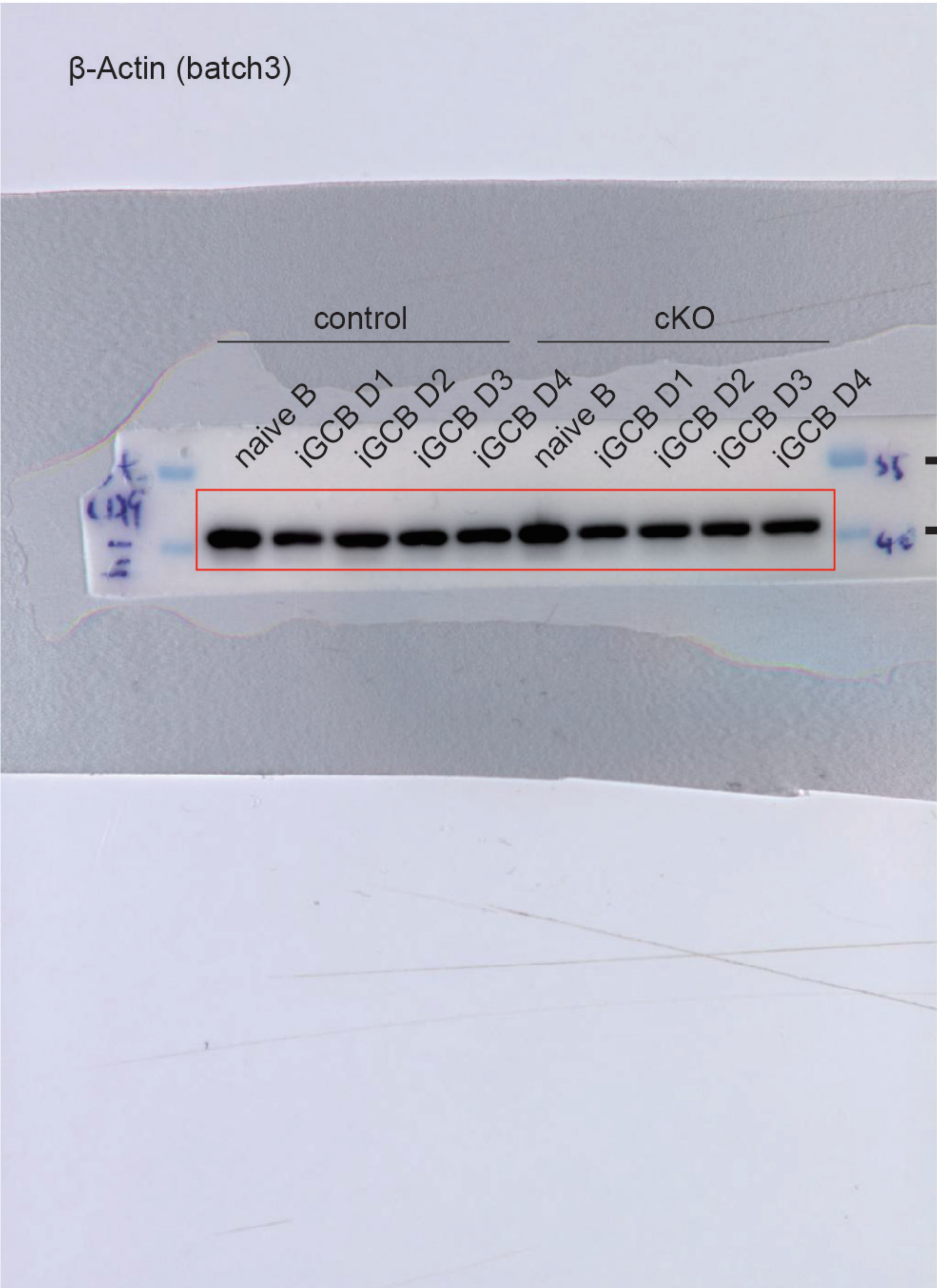

Supplement: SourceData FS3 — is the source file for Fig. S3. [file jem_20251968_sourcedatafs3.pdf]
